# A Reproducibility-Based Computational Framework Identifies an Inducible, Enhanced Antiviral State in Dendritic Cells from HIV-1 Elite Controllers

**DOI:** 10.1186/s13059-017-1385-x

**Published:** 2018-01-29

**Authors:** Enrique Martin-Gayo, Michael B. Cole, Kellie E. Kolb, Zhengyu Ouyang, Jacqueline Cronin, Samuel W. Kazer, Jose Ordovas-Montanes, Mathias Lichterfeld, Bruce D. Walker, Nir Yosef, Alex K. Shalek, Xu G. Yu

**Affiliations:** 1 Ragon Institute of MGH, MIT and Harvard, Cambridge, MA USA; 2 Department of Physics, University of California, Berkeley, CA USA; 3 Institute for Medical Engineering & Science (IMES) and Department of Chemistry, MIT, Cambridge, MA USA; 4 Broad Institute of MIT and Harvard, Cambridge, MA USA; 5 Infectious Disease Division, Brigham and Women’s Hospital, Boston, MA USA; 6 Howard Hughes Medical Institute, Chevy Chase, MD USA; 7 Electrical Engineering & Computer Sciences, UC Berkeley, Berkeley, CA USA; 8 Chan Zuckerberg Biohub, San Francisco, CA 94158 USA

**Keywords:** HIV-1, Dendritic cell, Single-cell RNA-seq, Single-cell genomics, Elite controller, Adjuvant, Reproducibility, Differential expression

## Abstract

**Background:**

Human immunity relies on the coordinated responses of many cellular subsets and functional states. Inter-individual variations in cellular composition and communication could thus potentially alter host protection. Here, we explore this hypothesis by applying single-cell RNA-sequencing to examine viral responses among the dendritic cells (DCs) of three elite controllers (ECs) of HIV-1 infection.

**Results:**

To overcome the potentially confounding effects of donor-to-donor variability, we present a generally applicable computational framework for identifying reproducible patterns in gene expression across donors who share a unifying classification. Applying it, we discover a highly functional antiviral DC state in ECs whose fractional abundance after in vitro exposure to HIV-1 correlates with higher CD4^+^ T cell counts and lower HIV-1 viral loads, and that effectively primes polyfunctional T cell responses in vitro. By integrating information from existing genomic databases into our reproducibility-based analysis, we identify and validate select immunomodulators that increase the fractional abundance of this state in primary peripheral blood mononuclear cells from healthy individuals in vitro.

**Conclusions:**

Overall, our results demonstrate how single-cell approaches can reveal previously unappreciated, yet important, immune behaviors and empower rational frameworks for modulating systems-level immune responses that may prove therapeutically and prophylactically useful.

**Electronic supplementary material:**

The online version of this article (10.1186/s13059-017-1385-x) contains supplementary material, which is available to authorized users.

## Background

Effective immune responses are founded upon the orchestrated dynamics of complex cellular ensembles. Over the past several decades, substantial work has been done to catalog the cell types, states, and interactions that inform these behaviors [1–7]. However, recent studies have shown that seemingly identical cell populations can exhibit significant and functionally relevant heterogeneities [1, 8–12]. While this unprecedented degree of cellular diversity challenges our understanding of the structure behind systems-level immune responses, it also presents new opportunities for identifying potential therapeutic or prophylactic strategies rooted in modulating immune composition and interactions.

One powerful approach for uncovering correlates of immune fitness is to study individuals that demonstrate exceptionally effective immune phenotypes [13], such as resistance to or immunological control of HIV-1 infection. Analysis of T cells from persons resistant to HIV-1 infection has linked genetic variation in the *CCR5* locus to reduced risk [14]. Similarly, studies of elite controllers (ECs)—a rare (~ 0.5%) subset of HIV-1 infected individuals who naturally suppress viral replication without combination antiretroviral therapy (cART) [15, 16]—have highlighted the importance of specific *HLA-B* variants and enhanced cytotoxic CD8^+^ T cell responses [17, 18]. Although compelling, these findings have proven insufficient to explain the frequency of viral control in the general population; additional cellular components or interactions could be implicated in coordinating effective host defense. Moreover, these studies have not suggested clinically actionable targets for eliciting an EC-like phenotype in other HIV-1-infected individuals. Further work has demonstrated improved crosstalk between the innate and adaptive immune systems of ECs [19–21]. For example, we recently reported that enhanced cell-intrinsic responses to HIV-1 in primary myeloid dendritic cells (mDCs) from ECs lead to effective priming of HIV-1-specific CD8^+^ T cell responses in vitro [20]. Nevertheless, the master regulators driving this mDC functional state, the fraction of EC mDCs that assume it, its biomarkers, and how to potentially enrich for it are unknown.

The recent emergence of single-cell RNA-sequencing (scRNA-seq) affords a direct means of identifying and comprehensively characterizing functionally important subsets of cells and their complex underlying biology. As scRNA-seq has matured into a mainstream technology, new questions about how to model single-cell variation continue to arise. To date, computational modeling approaches have typically described single-cell heterogeneity as a combination of gene-intrinsic effects (i.e. fundamental molecular noise), and gene-extrinsic ones, with the latter capturing both cell-intrinsic features (e.g. differences in intracellular protein levels, epigenetic state, mutation status, extracellular environment) and library-intrinsic technical artifacts (e.g. drop-out effects). Yet, in single-cell studies that utilize samples from across multiple donors (e.g. EC patients), these gene-extrinsic sources can be further subdivided into those that are unique to specific donors and those that are shared. The category of donor-dependent variation ranges from donor-specific cell subsets or large differences in cell-type composition to more subtle expression differences in constituent cell types. If the goal of a study is to generate hypotheses relating to a common phenotype, such as EC, strategies for prioritizing shared features can benefit from quantitative characterizations of reproducibility across multiple donors.

Here, we apply scRNA-seq to evaluate heterogeneity of transcriptional responses of mDCs (CD14^-^, CD11c^Hi^, HLA-DR^+^) from three EC individuals after in vitro exposure to a VSV-G pseudotyped HIV-1 virus or media control. To overcome the potentially confounding effects of donor-dependent biological and technical variation, we propose a broadly applicable strategy that combines reproducibility-based computational analyses with targeted experimentation to resolve, characterize, and modulate common response states across multiple donors (Additional file 1: Figure S1). More specifically, we utilize existing tools developed by our group for single-cell data analysis, including SCONE [22] and FastProject [23], and implement an irreproducible discovery rate (IDR)-based framework [24] in scRAD (Single-Cell Reproducibility Across Donors; https://github.com/YosefLab/scRAD) to identify reproducible response states, pathways, and biomarkers that are consistently detected after viral exposure across multiple donors who share a unifying classification such as EC. Our analysis reveals remarkable functional heterogeneity among mDCs, described by several discrete transcriptional response states. We discover one reproducible state that displays gene expression features consistent with profound functional activation and heightened antiviral activity. This subset of mDCs, enriched among cells expressing the surface molecules PD-L1 and CD64, is: (1) is induced more efficiently in ECs than in HIV-1 chronic progressors (CPs) or healthy donors (HDs) after in vitro viral exposure; (2) associated with both higher CD4^+^ T cell counts and lower HIV-1 viral loads; (3) more effective at stimulating T cell proliferation in vitro; and (4) more efficient at inducing HIV-1-specific polyfunctional cytotoxic CD8^+^ T cells—all canonical correlates of antiviral immunity in EC [25]. By leveraging *scRAD* to re-examine publicly available transcriptomic datasets, we further identify and experimentally investigate key regulatory molecules and adjuvants for modulating the acquisition of this functional mDC response state in the general population, with potential therapeutic and prophylactic implications. Together, our results highlight how single-cell analytic approaches can identify drivers of enhanced immunity and empower rational strategies for altering ensemble cellular responses. Notably, key analyses in this paper can be reproduced by following the *scRAD* package vignette (*scRAD Vignette*; https://github.com/YosefLab/scRAD/tree/master/vignettes).

## Results

### Shared EC mDC subsets revealed by scRNA-seq

**Fig. 1:**
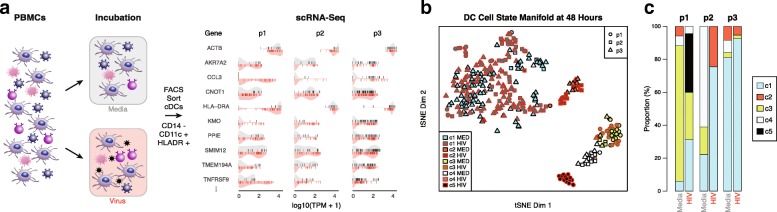
scRNA-seq identifies five response clusters among EC mDCs. **a** *Left*: *Schematic representation* of experimental system. After incubation with virus or a media control for 48 h, mDCs were isolated from PBMCs by FACS and profiled by scRNA-seq. *Right*: *Violin plots* of single-cell expression levels for ten select genes for each EC donor (p1, p2, p3). *Vertical lines* represent individual cellular values; the upper (*gray*) half of the violin shows the distribution of values for the media control and the bottom (*red*) shows the same for virus-exposed cells. **b** t-distributed stochastic neighbor embedding (tSNE) of all FACS sorted mDCs across three EC subjects passing quality filters (see “Methods”; p1: *circles*, p2: *triangles*, p3: *squares*). Virus exposed cells are outlined in *red*; media exposed cells have no outline. Cells separate into five distinct clusters (c1–5; see “Methods”). **c** *Stacked bar plot* depicting the percentage of total mDCs in each cluster for each patient under media and viral exposure conditions

In order to identify features of mDC (CD14^-^, CD11c^Hi^, HLA-DR^+^) innate immune responses to HIV-1 shared across ECs, we performed scRNA-seq [2, 9, 10, 26, 27] on peripheral blood mononuclear cells (PBMCs) from three ECs (p1, p2, p3) exposed in vitro to either a VSV-G pseudotyped HIV-1 virus or a media control for 48 h (Fig. 1a, see “Methods”) [28]. Stimulating PBMCs mimics some of the critical physiological interactions that occur between mDCs and other immune cell types, while the use of a VSV-G pseudotyped HIV-1 particles enhances mDC infection efficiency [29]. Given the potential bias of viability sorting, which may discard dying dendritic cell (DC) undergoing viral stress responses, we opted for in silico viability gating: following incubation, we sorted single mDCs and performed SMART-Seq2-based scRNA-seq [30]. After estimating gene expression levels, we applied elements of the *SCONE* [22] normalization pipeline to filter out single-cell samples with poor alignment characteristics and normalize the remaining data to minimize the impacts of these characteristics on expression quantification (Additional file 1: Figure S2, see “Methods”). Subsequently collected viability-sorted mDC data exhibited only a two- to threefold gain in the fraction of high-quality cells, suggesting that incubated primary cells from HIV-1 infected patients represent a fragile source material (Additional file 1: Figure S2). In total, we quantified high-quality expression levels in 188 virus- and 130 media-exposed cells by sequencing to an average depth of 700,000 reads (see “Methods”).

Low-dimensional representation of normalized expression estimates (see “Methods”) with t-distributed stochastic neighbor embedding (tSNE) illustrates how cells from each of the three EC donors span a common expression state-space: cells from different donors often share similar expression profiles, forming mixed clusters. Unsupervised k-medoids clustering revealed five distinct transcriptional response states (clusters 1–5 [c1–5]; Fig. 1b, see “Methods,” and Additional file 2: Table S1), with all but one state (c5) observed in all three donors. Linear regression analysis identified a small number of genes exhibiting significant cluster-independent associations with patient and exposure (131 and 14 genes, respectively). On the other hand, the fractional abundance of c1–c4 varied significantly across the three donors and two exposure conditions (see “Methods”). Among these, the c1 response state was consistently enriched among virally exposed mDCs (*p* value = 8.5 × 10^–6^, logistic regression, Wald test) while c3 and c4 were more common among media-exposed cells (*p* value = 1.3 × 10^–4^and 1.1 × 10^–5^, respectively, logistic regression, Wald test) (Fig. 1c). Similar, though less pronounced, shifts were observed for mDCs from donor p1 after 24 h (Additional file 1: Figure S3).

Within the virus-exposed p1 mDCs, we detected viral product (primed from adenine-rich regions in the pseudotyped HIV-1; see “Methods,” Additional file 1: Figure S4), allowing us to consider associations between cell intrinsic responses and viral sequences. Viral product was observed at comparable frequencies across the four “universal” clusters c1–c4 (not significant, Chi-squared test; data not shown), though we were limited to small numbers of cells (n_virus detected_ = 29) for this analysis.

Together, the above findings suggest that average virus-induced expression changes in the DC compartment are well explained by shifts in the frequencies of relatively invariant cell states.

### Reproducibility-based functional analysis reveals a robust antiviral signature among c1

**Fig. 2:**
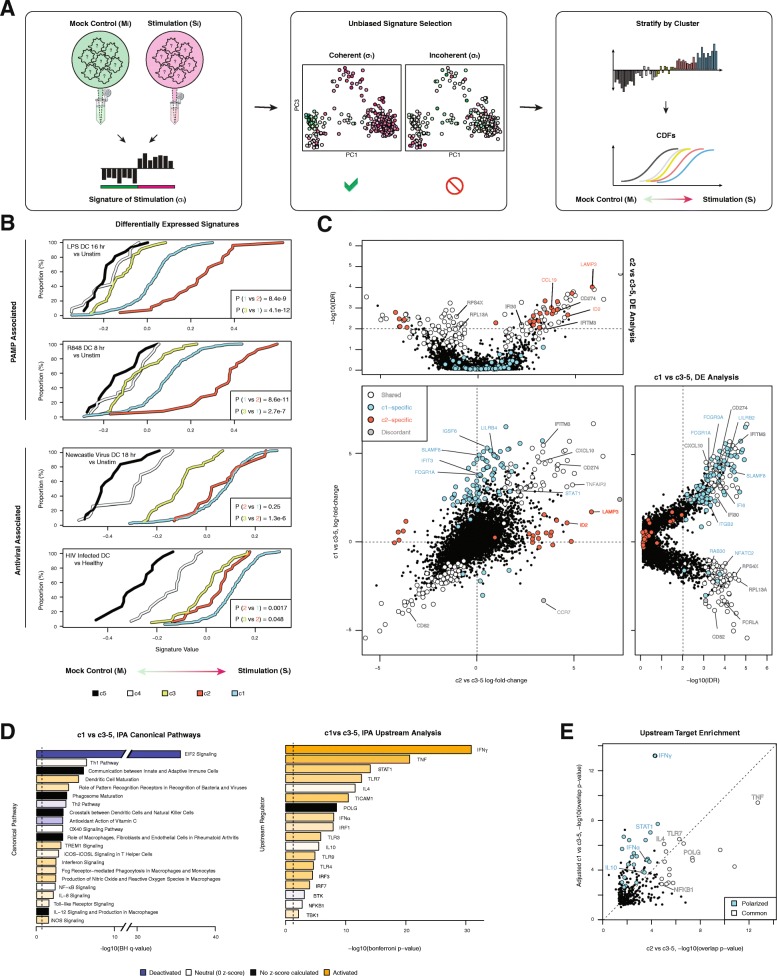
Characterization of transcriptional single-cell response groups. **a** *Left*: *Schematic* of signature database. The expression of a bulk sample of simulated DCs (S_i_) is compared to the expression of a mock control (M_i_). Highly ranked upregulated and downregulated genes comprise the signature ${\upsigma}_{\mathrm{i}}$. *Middle*: ${\upsigma}_{\mathrm{i}}$ is applied to all cells in the study and FastProject identifies pairs of expression data projections and ${\upsigma}_{\mathrm{i}}$ for which ${\upsigma}_{\mathrm{i}}$ varies coherently across the projection. *Right*: Coherent ${\upsigma}_{\mathrm{i}}$ values are binned by cluster to nominate specific cluster contrasts as biologically meaningful. **b** Cumulative distribution function (CDF) comparisons for single cells from each cluster identified in Additional file 1: Figure S1 with FastProject gene signatures derived from GSE14000 [57], GSE22589 [29], GSE18791 [58], and GSE2706 [59] (see “Methods”). The single-cell signature value quantifies the extent to which each cell is polarized toward a stimulated instead of unstimulated expression state. Clusters with gene expression signatures more closely mapping to the stimulated condition shift right, while clusters characteristic of unstimulated shift left. Kolmogorov–Smirnov (KS) tests show significant differences in these signatures between the first three clusters (c1, *n* = 220; c2, *n* = 26; c3, *n* = 35). **c** Potential genes specific for c1 (cyan), c2 (orange), shared between c1 and c2 (*white*) or inconsistent across individuals (*gray*). Individual volcano plots of negative log IDR vs mean differential log-expression between clusters c1 and c3–5 (*right*) and c2 vs c3–5 (*left*; see “Methods”). **d** Selected ingenuity pathway analysis (IPA) (see “Methods”) results for canonical pathways (Benjamini–Hochberg q value < 0.01) and upstream regulators (Bonferroni *p* value < 0.05) significantly deactivated (*blue*), neutral (*white*: with z score; *black*: without z score), or activated (*orange*) in c1 vs c3–5. **e** Comparison of putative upstream regulators from IPA for c1 vs c2–5 and c2 vs c3–5 (see “Methods”)

To further examine these five EC mDC response states and their inter-relationships, we utilized FastProject [23], a software package for visualization and interpretation of scRNA-seq data with reference to prior biological knowledge (see “Methods”). Coherently varying gene expression signatures identified by FastProject (Fig. 2a and Additional file 3: Table S2) repeatedly implicated c1 and c2, but not c3–c5, as responses associated with elevated DC activation (Fig. 2b and Additional file 1: Figure S5). Intriguingly, the transcriptional behavior of c1 mDCs appeared more consistent with elevated innate antiviral activity, displaying maximal values among signatures for DCs exposed to viruses, such as HIV-1 and Newcastle virus (*p* value = 2.5 × 10^–9^, 7.2 × 10^–13^, respectively; two-sided Kolmogorov–Smirnov (KS) test c1 vs c3; c1, *n* = 220; c2, *n* = 26; c3, *n* = 35; Fig. 2b). In contrast, c2 was well distinguished by signatures of DCs stimulated through alternative pathogen associated molecular patterns (PAMPs), such as LPS and R848 (*p* value = 8.4 × 10^–9^, 8.6 × 10^–11^, respectively; two-sided KS test c2 vs c1; c1, *n* = 220; c2, *n* = 26; c3, *n* = 35; Fig. 2b), or by bacteria or parasites (Additional file 1: Figure S5A). Motivated by the biological relevance of signatures contrasting c1 and c2 against the remaining clusters, we tested for differential expression (DE) of each of these two populations against the pool of c3, c4, and c5 cells.

As in most experiments involving non-model organisms, inter-subject biological and technical variability poses a substantial confounding risk by systematically distorting or exaggerating transcriptome-wide differences between groups. To address this, we developed and applied the DE module of *scRAD*: instead of explicitly modeling donor effects on single-cell expression distributions [31], *scRAD* performs DE analysis separately for every donor (or donor-pool) and then combines the results using IDR meta-analysis [24] (see scRAD vignette; see “Methods”). This model-based meta-analysis technique has demonstrated greater discriminative power than other approaches in simulation studies [24]; in our study, it better emphasizes aspects of clustering that are reproduced over multiple donors (Additional file 1: Figure S6). In order to partition differentially expressed genes (c1 vs c3–5 and c2 vs c3–5) into a common-evidence set from both clusters (c1 and c2) and two cluster-unique sets, we used *scRAD* again, this time performing meta-analysis to aggregate the DE results obtained independently for c1 and c2 (see “Methods”).

In line with known pathway elements shared between the DC antiviral and bacterial/parasitic response pathways [32, 33], we uncovered 121 genes that were commonly upregulated when comparing either c1 or c2 to c3–5 (Fig. 2c, Additional file 4: Table S3). Additionally, we identified 103 genes that were uniquely called as up- or downregulated in c1 or c2 relative to the remaining clusters (Fig. 2c). Genes preferentially expressed by c1 include the interferon-inducible gene *IFIT3*, whereas genes preferentially expressed by c2 encode molecules associated with endocytosis and antigen presentation (e.g. *LAMP3* [34], Fig. 2c), suggesting different levels of activation or polarization between c1 and c2. A targeted analysis of the expression of 28 interferon-stimulated genes (ISGs) regulated by HIV-1 [20, 35] suggested that c1 displayed the most potent and coherent interferon-induced transcriptional signatures (*p* value = 2.5 × 10^–7^, two-sided KS test c1 vs c2; c1, *n* = 220; c2, *n* = 26; Additional file 1: Figure S5). Interestingly, several canonical antiviral response genes were differentially expressed between virus- and media-exposed c1 cells, highlighting that stimulation-induced changes also contribute modestly to measured transcriptional variation (Additional file 1: Figure S4).

IPA [11] of differentially expressed gene lists revealed that the gene set reproducibly differentiating c1 from c3-5 is enriched for pathways related to DC maturation (Benjamini–Hochberg [BH] q value = 4 × 10^–6^), innate recognition of microbes by PRR (q = 8 × 10^–5^), interferon (q = 3 × 10^–3^) and TLR signaling (q = 0.03, Fig. 2d). These pathway enrichments do not reach significance for c2 (Additional file 1: Figure S5). We partitioned the set of putative upstream regulators predicted by IPA according to “common” or “polarized” activity across c1 and c2 (see “Methods”). Among the latter, we observed several molecules associated with antiviral responses with enhanced activity in c1 (IFNG, IFNA, STAT1). We also saw evidence of specific TLR activation (TLR3, TLR4) for c1 but not c2 (Fig. 2d, e and Additional file 1: Figure S5). Overall, these observations suggest that c1 represents a subset of mDCs in an activated viral response state that could potentially inform the effective innate antiviral immune responses observed in bulk mDC from ECs [20].

### Reproducible biomarker identification for c1 mDCs

To further study the c1 response state, we sought to identify putative markers for prospectively isolating c1 cells after exposure to HIV-1 across ECs. We developed two reproducibility-based criteria for surface marker candidacy, which have been implemented in the biomarker selection module of *scRAD*: (1) the surface marker must be encoded by a transcript that is reproducibly up-regulated in c1 vs c3–5 (IDR < 0.01); and (2) the transcript encoding the surface marker should be correlated with sufficiently many genes, in a reproducible manner, across all donors (Additional file 1: Figure S6; see “Methods” and scRAD vignette for additional details).

**Fig. 3:**
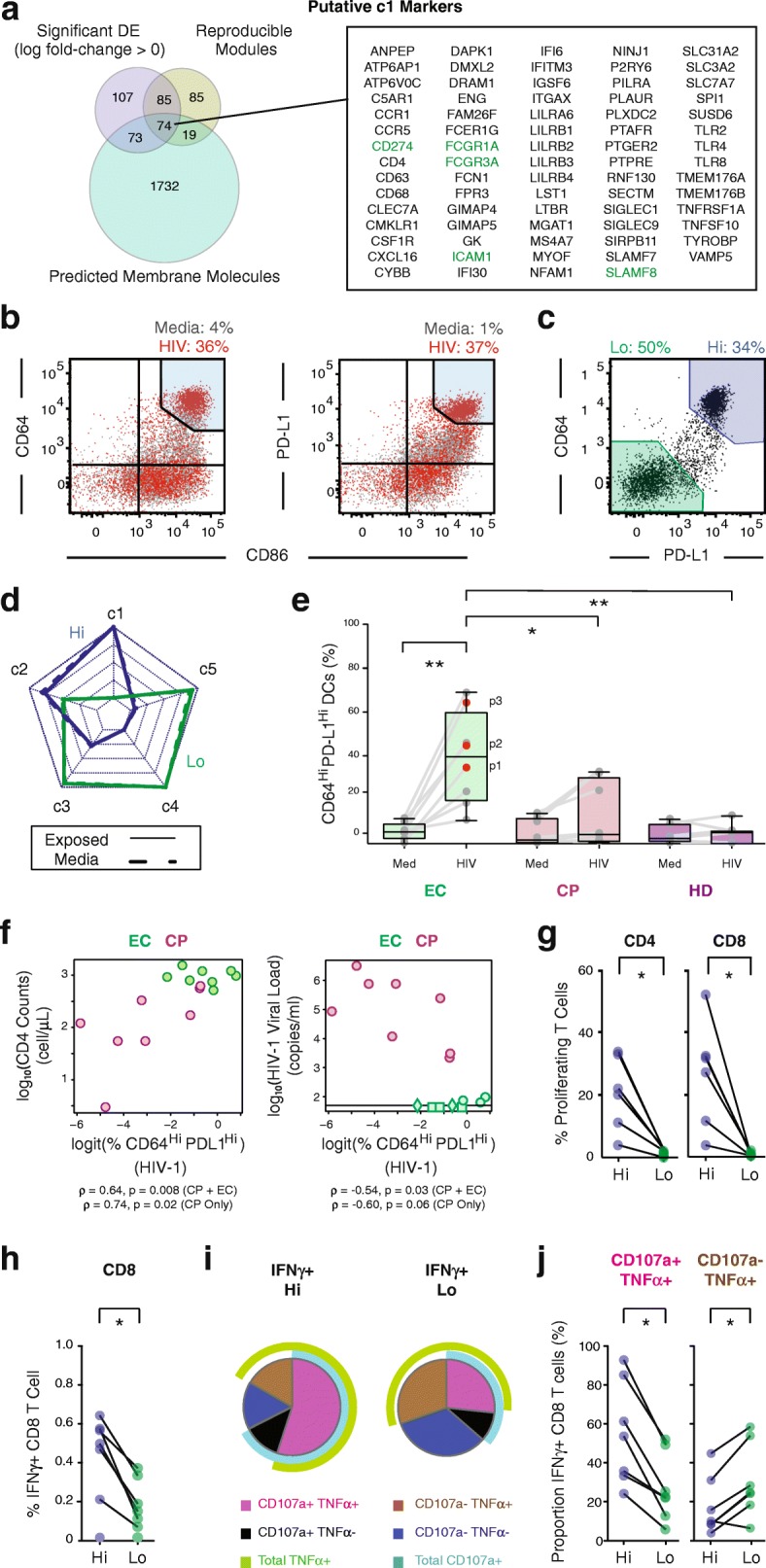
CD64 and PD-L1 enrich in highly functional c1-like mDCs. **a** Selection of c1-specific genes encoding surface proteins for validation as c1 markers. 74 genes (listed in box) were: (1) differentially expressed between c1 and c3–5; (2) reproducibly correlated with other c1 genes across all three ECs profiled; and (3) predicted membrane proteins (see “Methods”). Candidate markers shown in *green* were selected for validation by FACS (Fig. 2a, Additional file 1: Figure S7). **b** *Flow cytometry analysis* of either CD64 (*y-axis*, *left panel*) or PD-L1 (*y-axis*, *right panel*) vs CD86 (*x-axis*) expression in mDCs from EC patient 1 (p1). Numbers above represent the percentage of CD64^Hi^/PD-L1^Hi^ cells (*top right gate*; *light blue*) at 24 h in media (*gray*) and VSV-G pseudotyped HIV-1 virus exposure (*red*) conditions. **c** *Flow cytometry plots* showing analysis of CD64 vs PD-L1 expression on mDCs exposed to VSV-G pseudotyped HIV-1 for 24 h, defining two populations: CD64^Hi^,PD-L1^Hi^ (Hi; *blue*) and CD64^Lo^,PD-L1^Lo^ (Lo; *green*). Percentage in each gate is listed above. **d** *Radar plots* (see “Methods”) representing relative similarities of each subset (c1–5) to population-level RNA-Seq data from cells in the Hi and Lo PD-L1,CD64 gates 48 h after viral (*solid line*) or media exposure (*dashed line*). **e** Proportions of CD64^Hi^,PD-L1^Hi^ mDCs induced from multiple ECs (*n* = 8), untreated CPs (*n* = 8), and HDs (*n* = 7) after 24 h of culture in media or VSV-G pseudotyped HIV-1 (*, *p* < 0.05; **, *p* < 0.01; two-tailed Wilcoxon signed-rank test). **f** Correlation between the proportions of CD64^Hi^,PD-L1^Hi^ mDCs induced in ECs (*n* = 8) and untreated CPs (*n* = 8) or just CPs and clinical CD4 T cell count (*p* value = 8 × 10^–3^ [two-sided] and 2 × 10^–2^ [one-sided], respectively, Spearman correlation permutation *p* value) or between the proportions of CD64^Hi^,PD-L1^Hi^ mDCs induced in ECs (*n* = 8) and untreated CPs (*n* = 8) or just CPs and HIV-1 viral load (*p* = 3 × 10^–2^ [two-sided] and 6 × 10^–2^ [one-sided], respectively, Spearman permutation *p* value). *Diamond* and *square points* represent indeterminate viral loads of < 20 and < 50 copies/mL, respectively. **g** Proportion of proliferating CD4^+^ (*left*) and CD8^+^ (*right*) T cells co-cultured with the Hi and Lo sorted virus-exposed mDCs populations (*n* = 6 patients). **h** Proportion of total IFNγ^+^ CD8^+^ T cells cultured with the Hi and Lo sorted virus-exposed mDCs populations (*n* = 7 patients). Statistical significance for (**g**, **h**) were evaluated using a two-tailed Wilcoxon matched pairs signed-rank test (*, *p* < 0.05). **i** *Pie chart* generated with data from *n* = 7 patients showing CD107a and TNFα expression on CD8^+^ T cells cultured with Hi (*left*) or Lo (*right*) mDCs. **j** *Scatter plots* of proportions of CD107a^+^, TNFα^+^ (*left*) and CD107a^+^, TNFα^-^ (*right*) CD8^+^ T cells cultured with Hi and Lo mDCs. Statistical significance was evaluated using a two-tailed Wilcoxon matched pairs signed-rank test, *n* = 7 patients (*, *p* < 0.05)

Using this procedure, we obtained a list of 74 candidate c1 mDC markers (Fig. 3a). Based on antibody availability, we selected five proteins (*FCGR3*, *FCGR1*, *CD274*, *ICAM1*, *SLAMF8*) to profile 24 h after infection with pseudotyped HIV-1 by flow cytometry in CD14^-^ CD11c^Hi^ HLADR^+^ DCs from our cultures (Fig. 3b and Additional file 1: Figure S7). Importantly, the proportions of these cells and their expression of CD11c were similar after different durations of culture (Additional file 1: Figure S7A). Among these, both CD64 (*FCGR1A*) and PD-L1 (*CD274*) exhibited the most dramatic and consistent virus-induced upregulation among CD14^-^, CD11c^Hi^, HLA-DR^+^ mDCs isolated from the PBMCs of the three ECs characterized by scRNA-seq, as well as those from five additional EC donors (Fig. 3b; *p* value = 7.8 × 10^–3^; two-tailed Wilcoxon matched-pairs signed rank test; *n* = 8). CD64 is an Fc-receptor for IgG [36], while PD-L1 has been implicated in mediating the balance between T cell activation and immunopathology, as well as immediate effector differentiation and long-term memory formation in T cells [37]. Importantly, high expression of PD-L1 has also been found on tolerogenic murine mDCs in chronic LCMV infection [38] and in inflammatory lymph node-resident mDCs from HIV-1 infected individuals [39]. Nevertheless, high expression of IFN and inflammatory cytokines identified in our pathway analysis of c1 and high CD86 expression levels on CD64^Hi^ and PD-L1^Hi^ cells indicates that these cells are highly activated inflammatory DCs (Fig. 2 and Additional file 1: Figure S9D).

**Fig. 4:**
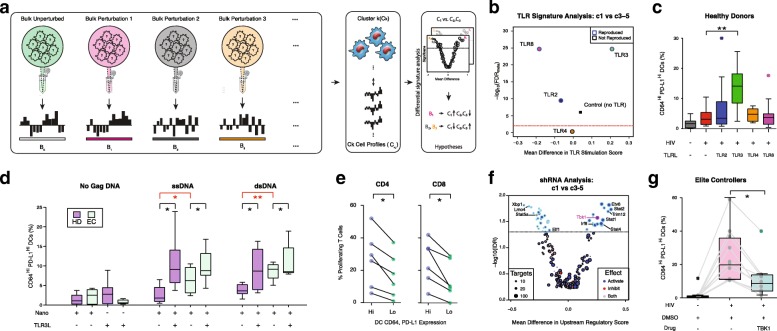
Immunomodulators can alter the fractional abundance of the c1 mDC phenotype. **a** *Top*: *Schematic* of bulk expression data (B_i_) from publicly available perturbation data. *Bottom*: Each cell’s expression profile (C_1j_) is correlated with all B_i_ so as to compare similarities of the single-cell cluster 1 to all bulk expression profiles. **b** *Volcano plot* of negative log meta-analysis false discovery rate (FDR) vs mean difference in “TLR stimulation score” between c1 and c3–5. Scores are computed from weighted correlations between single-cell profiles and transcriptional patterns from human DCs (see “Methods”) after 48 h of stimulation with media control (*black*) or agonists for either TLR2 (PAM3CSK4, *dark blue*), TLR3 (Poly I:C, *green*), TLR4 (LPS, *orange*), TLR7/8 (Gard, *purple*), or TLR9 (CpG, *light blue*). Tests reproduced with FDR < 0.01 in both stratified analyses are highlighted in *blue*. **c** Proportion of CD64^Hi^,PDL1^Hi^ cells among mDCs from PBMCs isolated from HIV-negative individuals cultured in the absence or the presence of VSV-G pseudotyped HIV-1, alone or in combination with TLR ligands (TLRL: TLR2L, PGNA, *n* = 11; TLR3L, Poly I:C, *n* = 11; TLR4L, LPS, n = 8; TLR8L, CL097, *n* = 11; Methods). Statistical significance was calculated using Kruskal–Wallis and Dunn’s tests (**, *p* < 0.01). **d** Proportions of CD64^Hi^, PD-L1^Hi^ cells among mDCs from healthy individuals (*indigo*) and elite controllers (*olive*) cultured in the absence or the presence of Poly I:C and polymer nanoparticles loaded with single-stranded (ss) or double stranded (ds) 100 nucleotide HIV-1 DNA (see “Methods”; *n* = 8, HIV negative individuals; *n* = 7, ECs). Statistical significance was calculated using either two-tailed Wilcoxon signed-rank test (*black*) or two-tailed Mann–Whiney test (*red*) to compare differences within or among patient groups, respectively (**, *p* < 0.01; *, *p* < 0.05). **e** Proportion of proliferating CD4^+^ or CD8^+^ T cells after culture with Hi or Lo mDC from a HD stimulated with TLRL3 and nanoparticles containing gag single-stranded DNA (*, *p* < 0.05; two-tailed Wilcoxon signed-rank test. *n* = 6). **f** *Volcano plot* of negative log IDR vs mean difference in upstream regulatory score between c1 and c3–5 based on single-cell correlations with short hairpin RNA-perturbation profiles from mouse DCs stimulated with LPS for 6 h (adapted from Chevrier et al. [32]; see “Methods”). The net effect (activate, inhibit, both) of each perturbation is denoted by color (*red*, *blue*, *gray*, respectively), as is its breadth (size). **g** Proportions of CD64^Hi^,PD-L1^Hi^ cells among EC mDCs cultured in the presence or absence of virus and DMSO (control, *magenta*) or BX795 TBK1 inhibitor (*cyan*; *n* = 10; see “Methods”). Statistical significance was calculated using a two-tailed Wilcoxon signed-rank test (*, *p* < 0.05)

When we analyzed mDCs based on surface expression levels of CD64 and PD-L1, we observed two dominant mDC populations after viral stimulation: one CD64^Hi^,PD-L1^Hi^ and the other CD64^Lo^,PD-L1^Lo^ (Fig. 4c). Population-level transcriptional profiling of mDCs sorted on CD64^Hi^,PD-L1^Hi^ at both 24 and 48 h post-viral stimulation revealed gene expression profiles dominated by the signature of the c1 and, to a lesser extent, c2 response states. In combination with the observation that mDCs sorted on CD64^Lo^,PD-L1^Lo^ matched a mixture of c3–5 (Fig. 3d), we concluded that CD64 and PD-L1 co-expression enriches for c1 cells. While these two markers are predominantly associated with c1 responses, we note that they are not necessarily causally involved in inducing either phenotype. In line with the single-cell observations above, both sorted mDC subsets exhibited similar levels of HIV-1 reverse transcription product at early time points after ex vivo infection (Additional file 1: Figure S7), suggesting differences in molecular sensing pathways, rather than viral replication dynamics, as the underlying driver of the different responses observed among c1-c4. Additionally, the c1-enriched/CD64^Hi^,PD-L1^Hi^ mDC phenotype observed in EC could be effectively induced in mDCs alone (without supporting PBMCs) exposed to VSV-G pseudotyped HIV-1 virus, indicating that generation of the CD64^Hi^,PD-L1^Hi^ mDC phenotype does not require paracrine signals from neighboring non-mDCs (Additional file 1: Figure S8). Collectively, these findings suggest that c1 mDCs might have the potential to drive enhanced antiviral antigen presentation relevant to control of HIV-1 infection.

### Functional characterization of c1 mDCs

Given the ties between strong antiviral activation and immune control of HIV-1, we naturally wondered whether the CD64^Hi^,PD-L1^Hi^ mDC phenotype, common to ECs, was uniquely enriched within these individuals and might be linked to common features of immune control against HIV-1. While this phenotype was consistently and efficiently induced in HIV-1 exposed mDCs from ECs, markedly lower proportions of it were observed in HIV-1 exposed mDCs from CPs and HDs (Fig. 3e; *n* = 8 per group). Consistent with the more effective induction of this phenotype in ECs, we found higher levels of type-I IFN present in culture supernatants from pre-isolated DCs exposed to HIV-1 from these patients as compared to alternative cohorts (Additional file 1: Figure S8B). Notably, these cohort-intrinsic differences were also observed when mDCs were exposed to a more physiological CCR5-tropic HIV-1 viral strain (Additional file 1: Figure S8; Additional file 5: Table S4), suggesting that this phenomenon is not restricted to VSV-G pseudotyped HIV-1 strains. Correlating the fractional abundance of CD64^Hi^,PD-L1^Hi^ mDCs after HIV-1 exposure against clinical phenotypes, we observed a significant positive association with CD4^+^ T cell count across both CPs (one-sided) and ECs + CPs (two-sided; *p* value = 2 × 10^–2^ and 8 × 10^–3^, respectively; Spearman correlation permutation *p* value). Plasma HIV-1 viral loads, meanwhile, were negatively associated with percentages of CD64^Hi^,PD-L1^Hi^ mDCs across all patients (*p* value = 3 × 10^–2^, Spearman correlation two-sided permutation p value), with borderline-significant association in CPs alone (*p* value = 6 × 10^–2^, Spearman correlation permutation one-sided p value, Fig. 3f). These associations show that a patient’s CD64^Hi^,PD-L1^Hi^ mDC fraction after viral stimulation tracks traditional biomarkers along a spectrum of HIV-1 control, suggesting that the ability to induce c1-like cells might be a useful biomarker of enhanced protective immune responses against HIV-1.

We next sought to directly probe the association between the induction of c1 responses and the enhanced functionality observed in bulk mDCs from EC. We first examined the putative enhanced antigen presentation and T cell activation abilities of the c1-like subset of mDCs by performing mixed leukocyte reactions to compare our CD64,PD-L1 high and low mDC subpopulations (see “Methods”). In these experiments, the c1-enriched/CD64^Hi^,PD-L1^Hi^ mDC population demonstrated superior ability to stimulate CD4^+^ and CD8^+^ T cell proliferation relative to CD64^Lo^,PD-L1^Lo^ mDCs across multiple ECs (Fig. 3g, Additional file 1: Figure S9, *p* value = 1.6 × 10^–2^ and *p* value = 3.1 × 10^–2^, respectively; two-tailed Wilcoxon matched-pairs signed rank test; n = 6). Similar results were observed in assays conducted with T cells from ECs, where CD64^Hi^,PD-L1^Hi^ mDCs were capable of efficiently stimulating the production of IFNγ in a significantly higher proportion of autologous CD8^+^ T cells as compared to CD64^Lo^,PD-L1^Lo^ mDCs (Fig. 3h; *p* value = 3 × 10^–2^; two-tailed Wilcoxon matched-pairs signed rank test; *n* = 5). Further, IFNγ^+^ CD8^+^ T cells primed in the presence of c1-enriched/CD64^Hi^,PD-L1^Hi^ mDCs expressed significantly higher levels of both the degranulation markers CD107a and TNFα (Fig. 3i,j and Additional file 1: Figure S9; *p* value = 1.5 × 10^–2^; two-tailed Wilcoxon matched-pairs signed rank test; n = 7), mirroring the polyfunctional CTL responses observed in ECs [17, 18]. We note that these differences are not significantly associated with disparities in HLA levels or viability among the CD64^Hi^,PD-L1^Hi^ and CD64^Lo^,PD-L1^Lo^ mDC subsets, but correlate with PD-L1 MFI levels in these populations (Additional file 1: Figure S9).

### Signature meta-analysis of candidate adjuvants for c1 mDCs

Given the possible therapeutic and prophylactic potential of c1-like DCs for studies in non-EC populations with less efficient responses to in vitro viral stimulation (Fig. 3e), we next sought to uncover the common signaling pathways involved in the acquisition of the c1-enriched/CD64^Hi^,PD-L1^Hi^ mDC phenotype so that we might engineer its frequency. IPA results for c1 had highlighted several signatures of human DC stimulation, including multiple components of several TLR signaling pathways (Fig. 2d; Additional file 1: Figure S5, Additional file 6: Table S5); thus, we aimed to compare our single-cell expression profiles to perturbed bulk expression data in order to determine which TLR pathways were most compatible with the c1 signature vs c3–5.

We define, for every cell and every TLR ligand we tested (see “Methods”), a “stimulation score,” which reflects the similarity between the cell’s transcriptional profile and the one induced by the ligand (using weighted correlation; see “Methods”). We then score each ligand by the extent to which its respective stimulation scores in c1 cells are higher than in clusters c3–5 (using a Kruskal–Wallis test). Finally, using the differential signature analysis module in *scRAD*, we combine the resulting *p* values across donors. Notably, for this analysis we used the Stouffer-Z *p* value combination method (Fig. 4a, see “Methods”) since the number of hypotheses (i.e. TLR ligands) is small, leading to instabilities in the IDR inference (see *scRAD* Vignette).

**Fig. 5:**
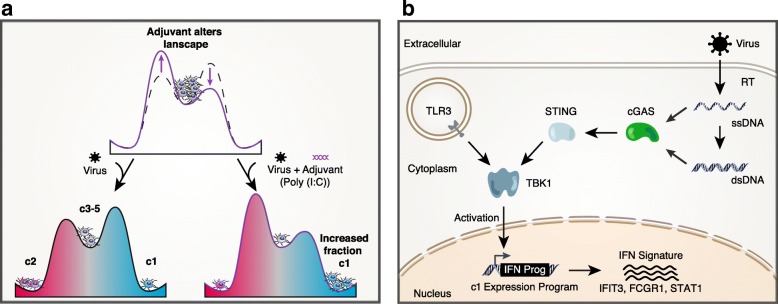
Unifying model of results. **a** Potential energy *diagram* conceptualizing how adjuvants and other perturbations alter the percentage of mDCs that enter the c1–5 response states upon viral or viral-like exposure. **b** Network *diagram* depicting tested nodes implicated in the c1 mDC response

Our meta-analysis showed that c1 cells correlated most positively with TLR3 stimulation via Poly I:C compared to the c3–5 (FDR < 0.01; Fig. 4b), generating the actionable hypothesis that triggering the endosomal dsRNA sensor TLR3 might selectively activate downstream pathways that synergize with innate viral sensing mechanisms to increase the fraction of mDCs maturing towards a c1-enriched/CD64^Hi^,PD-L1^Hi^ phenotype (Fig. 5). Analyses of microarray data from mouse DCs stimulated with a comprehensive panel of TLR ligands also suggested that the c1 state most strongly positively correlated with TLR3 activation [33] (see “Methods”; Additional file 1: Figure S10). To directly test this hypothesis, we incubated PBMCs from several HDs (*n* = 7)—which do not spontaneously generate significant numbers of c1-enriched/CD64^Hi^,PD-L1^Hi^ cells in vitro in the presence of VSV-G pseudotyped HIV-1 (Fig. 3e)—with virus and different TLR agonists for 24 h. In contrast to the other TLR ligands tested, we observed that co-incubation of mDCs with virus and Poly I:C led to a significant increase in the proportion of c1-enriched/CD64^Hi^,PD-L1^Hi^ mDCs in PBMCs from healthy individuals (TLR3L: *p* value = 0.0091, *n* = 11; Kruskal–Wallis and post-hoc Dunn’s test; TLR2L, TLR4L, and TLR8L, not significant; n = 11, 8, 11, respectively) (Fig. 4c). Similar results were observed after a longer 48 h incubation (Additional file 1: Figure S10). Meanwhile, in ECs, a TLR3, but not a TLR4, inhibitor had a modest, but significant, effect on the acquisition of the c1-enriched/CD64^Hi^,PD-L1^Hi^ mDC phenotype (*p* value = 3.9 × 10^–3^; two-tailed Wilcoxon signed-rank test (**, *p* < 0.01; *n* = 9; Additional file 1: Figure S10).

To explore the generality and therapeutic applicability of our adjuvant strategy, we next examined whether we could couple the same TLR3 activation with direct DNA-based targeting of the cytosolic innate immune recognition machinery that senses viral DNA products [40] rather than use the virus itself. To address this, we incubated PBMCs from HDs or ECs simultaneously with a TLR3 agonist (Poly I:C) and single- or double-stranded HIV-1 Gag DNA (ssDNA or dsDNA, respectively) encapsulated in polymeric nanoparticles (see “Methods”). A similar delivery vehicle has previously been shown to selectively activate cGAS- and STING-dependent immune recognition pathways, which are involved in innate immune sensing of HIV-1 during natural infection [41]. When we analyzed the fraction of mDCs differentiating into c1-enriched/CD64^Hi^,PD-L1^Hi^ cells, we found that activation with either ss/dsDNA or Poly I:C (TLR3 agonist) alone in PBMCs from HDs was less efficient at inducing c1-enriched responses (*p* value = 7 × 10^–2^, nano vs Poly I:C alone; *p* value = 5 × 10^–2^, nano vs ssDNA; *p* value = 1 × 10^–2^, nano vs dsDNA; two-tailed Wilcoxon matched-pairs signed rank test; *n* = 8; Fig. 4d, comparisons not highlighted). Combining both stimuli, however, significantly increased the proportion of c1-enriched/CD64^Hi^,PD-L1^Hi^ mDCs in PBMCs isolated from HDs (*p* value = 1.6 × 10^–2^ and *p* value = 3.1 × 10^–2^ for ss- and dsDNA, respectively; two-tailed Wilcoxon matched-pairs signed rank test; *n* = 8; Fig. 5d). Similar results were obtained with cells from ECs (*p* value = 0.0469 for both ss- and dsDNA; two-tailed Wilcoxon matched-pairs signed rank test; *n* = 7; Fig. 5d), with the notable exception that, in ECs, exposure to dsDNA alone led to significantly higher levels of c1-like/CD64^Hi^,PD-L1^Hi^ mDCs relative to cells cultured only in media (*p* value = 3 × 10^–2^; Wilcoxon matched-pairs signed rank test; *n* = 7; Fig. 5d, comparison not highlighted), suggesting a heightened baseline predisposition of EC to respond to intracellular DNA. In mixed leukocyte reactions, the CD64^Hi^,PD-L1^Hi^ mDCs generated from HDs incubated with TLRL3 and nanoparticles containing gag dsDNA stimulated greater proliferation in CD4^+^ and CD8^+^ T cells compared to the CD64^Lo^,PD-L1^Lo^ mDCs from the same assay (*p* value = 3.5 × 10^–2^ and *p* value = 3.1 × 10^–2^, respectively; two-tailed Wilcoxon signed-rank test; *n* = 6), suggesting that adjuvant induced CD64^Hi^,PD-L1^Hi^ mDCs in HDs are highly functional antigen presenting cells like their EC counterparts (Fig. 5e).

### Reproducible differential signature analysis reveals immunomodulators of c1 mDCs

To identify additional nodes for rationally modulating the acquisition of the c1 functional state, as well as to examine the general applicability of the IDR-framework for uncovering putative regulators of c1’s (or any other state’s) induction, we again applied the differential signature module of *scRAD* (see scRAD Vignette); in this instance, due to limited public availability of human perturbation data, we turned to a published dataset of the transcriptional effect of ~ 200 transcription factor and signaling molecule perturbations in LPS-stimulated mouse DCs that are highly conserved with humans [32, 33]. We ranked the perturbations by the degree to which they reproducibly favored the generation of one or more (here, c1) responses over others (here, c3–5; see “Methods”). Unlike in the TLR analysis, here we had a sufficient number of hypotheses, and therefore utilized *scRAD*’s core IDR-based functionality [24]. The resulting meta-analysis nominated several putative regulators for modulating the fractional abundance of c1 mDCs in response to a virus or virus-like stimulation (Fig. 4g). Among our top positive regulators of c1 was TBK1, a recognized signal mediator that is activated downstream of multiple innate immune sensing pathways at the convergence of the organelle-associated adaptors MAVS, TRIF (downstream effector of TLR3, TLR4), and STING (effector of the intracellular DNA sensor cGAS) [42–44], some of which were previously detected in our IPA Upstream Analysis (Fig. 4d). Notably, the cGAS-STING pathway is known to play a key role in the recognition of cytoplasmic HIV-1 DNA in myeloid cells, including those from ECs [20, 40], and cGAS itself (*MB21D1*) was upregulated in c1 cells (LFC = 1.9, IDR < 0.05). To evaluate whether signaling through TBK1 significantly contributes to the maturation of mDCs into the c1-enriched/CD64^Hi^,PD-L1^Hi^ subset in ECs, we added BX795, a TBK-1 antagonist, to PBMCs from ECs at the time of viral addition and examined the impact on mDC responses (see “Methods”). As shown in Fig. 4h, inhibition of TBK1 during viral exposure led to a dramatic and significant abrogation of the induction of the c1-enriched/CD64^Hi^,PD-L1^Hi^ mDC population in ECs (*p* value = 2.0 × 10^–3^; two-tailed Wilcoxon signed-rank test; *n* = 10), suggesting that TBK1 is a key driver of the acquisition of the c1 phenotype in mDCs and validating the promise of our computational framework.

## Conclusions

In summary, by studying elite immune control of HIV-1 infection as an example of enhanced immunity with a reproducibility-based framework that identifies gene expression features shared across patients that are linked by a common attribute, we identified a highly functional CD64^Hi^,PD-L1^Hi^ mDC response state that is primed to drive adaptive immunity—a previously unrecognized correlate of effective antiviral response against viral stimuli. Extending and developing computational approaches to hypothesize reproducible biomarkers and upstream regulators, we have realized a rational, extendable framework for modulating the relative abundance of this state. These tools, provided as part of the new R package, *scRAD*, can be applied to a wide variety of common scRNA-seq analyses and derive robustness from a reliance on multiple donors. An important feature of the IDR framework [24] is that it is based on rank transformed data rather than the original signal (e.g. *p* values); this facilitates the statistical analysis of reproducibility in any ranked set of hypotheses, beyond the three analysis modules presented here (DE, biomarkers, and upstream regulators). Notably, while the original IDR R-package accounts only for a two-donor scenario, our extension (see “Methods” and *scRAD* Vignette) facilitates meta-analysis of common signals across larger numbers of donors.

The heterogeneity of mDC responses identified in our study should invoke recent work by Villani et al. [7] that describes at least four subsets of circulating mDC in HDs. Interestingly, our c1-mDC response state shares important characteristics with the DC4 (CD11c^+^MHCII^+^CD1C^-^CD141^-^CD16^+^) subset described in that work, exhibiting its characteristic anti-viral signature as well as reproducible up-regulation of all five published marker genes [7]. Given the dissimilarities between cohorts and experimental conditions, future studies will be required to fully elucidate the functional and transcriptional relationships between these mDC groups and their ontogeny.

Importantly, our study demonstrates a clear association between the ability of ECs to efficiently acquire the CD64^Hi^,PD-L1^Hi^ mDC phenotype in vitro and clinical parameters of immunological control of HIV-1 infection. This suggests that an increased ability to induce the c1 transcriptional programs in mDCs might be indicative of beneficial immune responses associated with control of HIV-1 replication in ECs. An important limitation of our study design is that it only establishes associations, rather than causal relationships, between our observations and clinical and immunological parameters, i.e. it does not directly demonstrate a role for mDC in driving or promoting immune control of HIV-1 infection in ECs in vivo. Future studies will also be needed to directly examine the role of c1 DCs in other lymphoid tissues, such as lymph nodes, since our current work focused on PBMCs. Were this to prove true, our adjuvanting and perturbation experiments suggest exciting therapeutic possibilities for non-ECs via co-stimulation of TLR and DNA sensor agonists, and perhaps TBK1 directly. Intriguingly, high expression of PD-L1 has also been described on a subset of lymph node-resident mDCs from HIV-1 infected individuals spanning a range of viral loads [39]. While this study proposes that the lymph node resident PD-L1^+^ DC subset may dampen immune responses based on PD-L1 expression, as CD64 co-expression was not measured, the relationship of this state to c1 remains unknown. PD-L1 has also been associated with an alternative, tolerogenic IL-10-producing mDC population induced under long-term and chronic infection settings in mice [38]; this state is fundamentally distinct from the highly activated CD64Hi PD-L1Hi DC subset identified in our study which is characterized by expression of multiple inflammatory molecules (Fig. 2), high levels of activating costimulatory molecules (Fig. 3, Additional file 1: Figure S7 and Figure S9), and efficiently induces T cell proliferation and polyfunctionality. In general, the putative functional differences between the two states highlights the importance of surveying complete extra- and intra-cellular states in ascribing function, given potential redundancy. A critical limitation is the lack of an equivalent in vivo system where a direct and causal relationship between mDC responses and the induction of protective HIV-1-specific adaptive immunity can be safely and ethically tested; nevertheless, similar principles may inform future experiments performed with other viruses or virus-like elements (e.g. in a vaccine) in both humans and other organisms.

Mechanistically, further investigation will be required to identify what biases mDCs from ECs to respond at higher frequency with a c1-enriched phenotype. Given our adjuvanting and perturbation experiments, this enhanced antiviral response capacity could derive from variations in the basal abundance of different DC subsets which, in turn, each have an unequal propensities to generate the c1 responses to nucleic acids; it could similarly derive from dissimilarities in the intrinsic response properties of one or more progenitor or terminally differentiated states, informed by a combination of EC-specific epigenetic modifications and/or complex sets of genetic variants. Since our experimental validations support an inferred role for TLR3 in synergizing with cytosolic viral recognition machinery to induce a TBK1-dependent c1-enriched/CD64^Hi^,PD-L1^Hi^ response, we propose that simultaneous induction of DNA and dsRNA sensing through the cGAS-STING [20, 42] and TLR3 pathways might potentiate (Fig. 5a) the maturation (or selective survival) [45] of c1-enriched/CD64^Hi^,PD-L1^Hi^ cells by converging on TBK1 (Fig. 5b) and that these elements might be a natural nexus to explore for EC-specific molecule features. Still, our work demonstrates the potential of scRNA-seq to discover, genome-wide, functional cellular immune response states, associated markers, and shifts in abundance that may inform the overall efficacy of host immunity.

## Methods

### Study participants

HIV-1 elite controllers (ECs) who had maintained < 2000 copies/mL HIV-1 viral load (VL; 20–98 copies/mL, median 48 copies/mL) for a median of five years (range = 2–14 years) in the absence of antiretroviral therapy (EC; CD4^+^ T cell counts: 515–1543 cells/mL, median 909 cells/mL; *n* = 8 persons), untreated CPs (VL: 2190–3,117,608 copies/mL, median 162,807 copies/mL; CD4^+^ T cell counts: 3–623 cells/mL, median 146.5 cells/mL; *n* = 8 persons), and HIV-1 seronegative HDs (Neg; *n* = 7 persons) were recruited for this study (Additional file 5: Table S4). All individuals gave written informed consent; the Institutional Review Board of Massachusetts General Hospital/Partners Healthcare approved the study protocol.

### In vitro infection with HIV-1 virus

Freshly isolated PBMCs were infected with GFP-encoding vesicular stomatitis virus G envelop (VSV-G) pseudotyped or R5-tropic HIV-1 virus (multiplicity of infection [MOI] = 2.4 and 0.4, respectively), kindly provided by Dr. Dan Littman (New York University, New York, NY, USA), for 2 h at 37 °C. At 24 and 48 h post-infection, CD14^-^,CD11c^Hi^,HLADR^+^ mDC were singly sorted (see “Flow cytometric analysis and sorting”) from total PBMC into 96-well plates containing lysis buffer for scRNA-seq as previously described [46] (Fig. 1a). In some experiments, sorted CD14^-^ CD11c^Hi^ HLADR^+^ mDCs were presorted before ex vivo infection with VSV-G pseudotyped HIV-1.

### TLR perturbations

In the TLR agonist experiments, mDCs from PBMCs (see “Flow cytometric analysis and sortingg”) were treated with HIV-1 alone or HIV-1 in combination with 2 μg/mL of a TLR2 (PGNA), TLR3 (Poly I:C), TLR4 (LPS), or TLR8 (CL097) ligand (InvivoGen, San Diego, CA, USA) (Fig. 4c, Additional file 1: Figure S10). In the TLR antagonist studies, mDCs from PBMCs were treated with VSV-G pseudotyped HIV-1 (see “In vitro infection with HIV-1 virus”) alone or in combination with a TLR3 (CUCPT4A, 60 nM, Tocris), TLR4 (600 ng/mL, LPS-RS, InvivoGen), or TBK-1 inhibitor (BX795, 1 μM, InvivoGen) (Fig. 4b, g, Additional file 1: Figure S10).

For our ssDNA and dsDNA stimulation experiments (Fig. 4d), mDCs from either HDs (*n* = 8) or ECs (*n* = 7) were cultured for 24 h in the presence of Poly I:C and 2 μg/mL of either ss- or ds-Gag DNA [41] that had been encapsulated into polymeric nanoparticles (TransIT-X2, Myrus) following the manufacturer’s instructions. Importantly, this approach has been shown to enable intracellular delivery of nucleic acids in primary human innate immune cells, overcoming a critical barrier for nucleic acid delivery and sensing [47].

In our human TLR stimulation experiments (Fig. 4b), whole PBMCs from a HD were incubated for 48 h with or without 2 μg/mL of a TLR2 (PGNA), TLR3 (Poly I:C), TLR4 (LPS), or TLR8 (CL097) ligand (InvivoGen, San Diego, CA, USA). Following incubation, mDCs were sorted (see “Flow cytometric analysis and sorting”) into two replicate 5000–10,000 cell populations and sequenced (see “Single-cell and population RNA samples”).

### Flow cytometric analysis and sorting

PBMCs were stained with LIVE/DEAD cell blue viability dye (Invitrogen, Carlsbad, CA, USA) and monoclonal antibodies directed against CD11c (BioLegend, San Diego, CA, USA), CD14 (BD Biosciences, San Jose, CA, USA), HLA-DR, CD64, PD-L1, ICAM1, CD16, SLAMF8 (BioLegend) and subsequently analyzed on a Fortessa cytometer (BD Biosciences). Data were analyzed with FlowJo software (Tree Star, Ashland, OR, USA). mDCs were identified from bulk PBMCs as a population of viable CD14^-^ cells expressing high levels of CD11c and HLA-DR.

For the functional studies on mDC subsets, BVD-negative CD14^-^ CD11c^+^ HLADR^+^ mDCs were sorted into two subpopulations expressing high and low levels of CD64 and PD-L-1 (Fig. 3c).

In the experiments evaluating polyfunctional CD8^+^ T cell responses in EC, cultured cells (see “Activation of CD8^+^ T cells from EC with autologous CD64,PD-L1 Mdc”) were first labeled with LIVE/DEAD cell blue viability dye and anti-CD8 and CD-3 monoclonal Abs (BioLegend, San Diego, CA, USA). Subsequently, T cells were fixated and permeabilized and incubated with monoclonal antibodies against TNFα, IFNγ (BioLegend) and CD107a (BD Biosciences).

### Mixed leukocyte reaction assays

FACS purified viable CD64^Hi^,PD-L1^Hi^ and CD64^Lo^,PD-L1^Lo^ mDC subpopulations, generated after 24 h of infection with a VSV-G HIV-1 virus or 24 h of incubation with TLR3 ligands (2 μg/mL poly I:C and nanoparticle-loaded gag-dsDNA adjuvants), were mixed with allogeneic total peripheral blood T lymphocytes previously stained with 5 μM carboxyfluorescein succinimidyl ester (CFSE, Invitrogen) at a T:DC ratio of 4:1. As a control, T cells were also cultured in the presence of media alone or 2.5 μg/mL PHA (Sigma) and 50 IU/mL IL-2 (NIH AIDS reagent program). After incubation for six days, cells were washed, stained with viability dye and anti-CD4 and anti-CD8 antibodies (BioLegend, San Diego, CA, USA), and CFSE dilution on CD4^+^ and CD8^+^ T cell subpopulations was analyzed by flow cytometry using a Fortessa flow cytometer.

### Autologous CD64, PD-L1 mDC subsets

Total CD8^+^ T cells were isolated by magnetic cell sorting (DynaBeads, Thermo Fisher) from unstimulated PBMC from ECs (*n* = 5) and cultured in the absence or the presence of autologous CD64^Hi^,PD-L1^Hi^ and CD64^Lo^,PD-L1^Lo^ mDC sorted from an alternative PMBC culture previously infected with a VSV-G pseudotyped HIV-1 virus for 24 h, as previously described (see “In vitro infection with HIV-1 virus”) at a ratio (T:DC = 4:1). After 2 h of incubation, cultures were supplemented with Brefeldin A (BioLegend) and Monensin (BD-Biosciences) and left in culture for an additional 16 h. Phenotypic analysis of Polyfunctional CD8^+^ T cell responses was determined by flow cytometry of intracellular expression of IFNγ, TNFα and CD107a (see “Flow cytometric analysis and sorting”; Fig. 3h–j, Additional file 1: Figure S9).

### Quantification of HIV-1 by qPCR

HIV-1 reverse transcripts present in sorted mDC populations were amplified from cell lysates at 24 h post-infection as previously described [48]. Copy numbers of reverse transcripts were obtained after extrapolation to specific standard curves generated from HIV-1-infected 293 T cells [20]. qPCR data were normalized to relative *CCR5* gene copy number.

### Statistics of in vitro functional assays

The significance of differences in the fractional abundance of sorted mDC subsets across different cohorts and in our functional assays—including the mixed leukocyte reactions, culture of CD8^+^ T cells from EC with autologous mDC and the TLR ligand and DNA stimulation assays—were determined using two-tailed Wilcoxon matched-pairs signed-rank test. In some experiments, we applied a Kruskal–Wallis test with post-hoc Dunn’s test—adjusting for test multiplicity—using GraphPad Prism 6 software. The specific test used for each comparison is noted in the text.

### Single-cell and population RNA samples

Following sorting, whole transcriptome amplification (WTA) was performed on 96-well plates of single cells as described previously [46]. Briefly, individual lysed cells were cleaned with 2.2× volume AMPure XP SPRI beads (Beckman Coulter, Danvers, MA, USA) and isolated cellular messenger RNAs (mRNA) were reverse transcribed and amplified.

For the population samples, total RNA was isolated using a RNeasy plus Micro RNA kit (Qiagen, Hilden, Germany) following the manufacturer’s instructions. A total of 2 μL of this isolated RNA was then added to 8 μL of water and cleaned with 2.2× volume beads. Finally, 1 μL of this cleaned RNA was used in a WTA reaction [46].

Following WTA, PCR products were cleaned with 0.9× volume SPRI beads and eluted in water. The concentration of complementary DNA (cDNA) in the resulting solution was determined using a Qubit 3.0 Fluorimeter (ThermoFischer, Waltham, MA, USA) and analyzed using a high sensitivity DNA chip for BioAnalyzer (Agilent, Santa Clara, CA, USA).

### cDNA library preparation for RNA-seq

WTA products were diluted to a concentration of 0.1 to 0.4 ng/μL, tagmented and amplified using Nextera XT DNA Sample preparation reagents (Illumina, San Diego, CA, USA). Tagmentation was performed according to manufacturer’s instructions, modified to use one-quarter of the recommended volume of reagents, extending tagmentation time to 10 min and extending PCR time to 60s. PCR primers were ordered from Integrated DNA Technologies (Coralville, IA, USA). Nextera products were then cleaned twice using 0.9× SPRIs and eluted in water. The final library was quantified using Qubit and analyzed using a high sensitivity DNA chip. It was then diluted to 2.2 pM and sequenced on a NextSeq 500 (Illumina).

### Single-cell expression quantification

RNA-seq reads were aligned to the RefSeq hg38 transcriptome (GRCh38.2) using Bowtie2 [49]. The resulting transcriptomic alignments were processed by RSEM to estimate the abundance (expected counts and transcripts per million [TPM]) of RefSeq transcripts [50].

Several genes were quantified multiple times due to alternative isoforms unrelated by RefSeq annotation. Before expression data normalization, these TPM estimates were summed to produce a single TPM estimate per RefSeq gene symbol.

### Single-cell filtering and gene filtering

For each single-cell library, we computed transcriptome alignment and quality metrics using FastQC (Babraham Bioinformatics), Picard tools (Broad Institute), and custom scripts. Computed metrics included: (1) number of reads; (2) number of aligned reads; (3) percentage of aligned reads; (4) number of duplicate reads; (5) primer sequence contamination; (6) average insert size; (7) variance of insert size; (8) sequence complexity; (9) percentage of unique reads; (10) ribosomal read fraction; (11) coding read fraction; (12) UTR read fraction; (13) intronic read fraction; (14) intergenic read fraction; (15) mRNA read fraction; (16) median coefficient of variation of coverage; (17) mean 5’ coverage bias; (18) mean 3’ coverage bias; and (19) mean 5’ to 3’ coverage bias.

We used the *metric_sample_filter* function from the SCONE package [22] to flag libraries with low numbers of aligned reads (< 28,840), low percentages of aligned reads (< 15%), and low percentages of detected transcripts (< 33.4% of Ensembl GRCh38.80 protein-coding genes expressed at > 100 TPM in at least 10% of samples – or “common genes”) (Additional file 1: Figure S2A–C). We identified 99 genes of candidate constitutive expression by fitting a population-wide Fano factor as a linear function of mean TPM, selecting the 99 common genes with minimal fit residual. These genes covered a range of 50.0–35,000 TPM. For each sample, the relationship between mean detected TPM and detection rate (or “false-negative characteristic”) was modeled as a logistic function; the area under this fitted curve was utilized to distinguish samples with poor detection properties (Additional file 1: Figure S2D, E). Out of 2489 initial samples, only 393 (318 at 48 h and 75 at 24 h) samples passed this primary filter. We note that some of this loss is due to our decision to exclude viability stain for some scRNA-seq sorts. Importantly, this viability selection did not appear to skew the sub-composition of cell states passing our sample filtering criteria (see “Clustering analysis and visualization”).

Following cell filtering, genes were retained for downstream analysis if they were annotated as protein-coding and expressed at levels > 100 TPM in at least five high-quality cells.

### Single-cell data normalization

In order to normalize TPM data, we applied the full-quantile normalization method, restoring original zero values to zero following normalization. This restoration step was necessary due to widespread zero-ties. We used normalization metrics of the SCONE [22] package to assess performance of this strategy.

The first three scores measure the maximum absolute correlation between the first three principal components (PCs) of the TPM matrix and the first three PCs of: (1) the matrix of library-level qc metrics; (2) the un-normalized matrix of TPM estimates for “negative control” genes from the MSigDB^9^ “HSIAO_HOUSEKEEPING_GENES” gene set; and (3) the un-normalized matrix of TPM estimates for “positive control” genes from the MSigDB “REACTOME_INNATE_IMMUNE_SYSTEM” gene set. Following normalization, the first two scores decreased while the third increased slightly, suggesting that technical structure has been removed from the data while retaining structure associated with the biological processes at hand (Additional file 1: Figure S2F).

The next three scores measure the average silhouette width for various classifications across a Euclidean metric defined on the first three PCs of the TPM matrix: (1) biological class = patient ID × exposure × time point × viability; (2) batch class; and (3) average silhouette width where each stratification of batch and biology has been separately clustered using the partitioning around medoids (PAM) clustering algorithm. Following normalization, the first two scores decrease, suggesting that confounding by biological and batch factors could not be addressed by this normalization. However, the rise of the third score suggests greater intra-stratum clustering following normalization (Additional file 1: Figure S2G).

The last two scores—(1) the median absolute relative log-expression (RLE) and (2) the variance of the RLE interquartile range—both decreased, implying reduced global DE following normalization (Additional file 1: Figure S2H).

### Clustering analysis and visualization

Principal component analysis (PCA) was applied to all filtered and normalized single-cell log-TPM data collected at the 48-h time point; consequent analysis was limited to the first 50 PC values (defined per cell) explaining 32% of expression variance. For each choice of dimension d, in the range of 2–50, a Euclidean cell-distance matrix was computed over the first d PCs. The PAM clustering algorithm was used to cluster cells over a range of k = 2–10 clusters. Let S(k,d) represent the average silhouette width of a PAM k-clustering on d dimensions. We define k(d) as the unique choice of k that maximizes S(k,d) for any choice of d. We selected d so as to maximize cluster number and tightness:

$$ {\displaystyle \begin{array}{l}k(d)\ge k\left({d}^{\hbox{'}}\right)\forall {d}^{\hbox{'}}\ne d\\ {}S\left(k(d),d\right)\ge S\left(k\left({d}^{\hbox{'}}\right),{d}^{\hbox{'}}\right)\forall \left\{{d}^{\hbox{'}}|k\left({d}^{\hbox{'}}\right)=k(d)\right\}\end{array}} $$

d = 7 and k = 5 were the selected clustering parameters. This method is implemented in the *pamkd* function in the *scRAD* package.

Due to the high-dimension of the underlying expression space, clustering was visualized using a two-dimensional (2D) tSNE projection applied to the d = 7 distance metric (5000 iterations). The five clusters were annotated in clockwise order.

After clustering, we applied ordinary least-squared regression to model each gene i’s expression in cell j as a function of patient, exposure, and cell type:

$$ {g}_{ij}\sim {\alpha}_i\kern0.5em +\kern0.5em {\beta_i^p}^{\ast } Patien{t}_j\kern0.5em +\kern0.5em {\beta_i^e}^{\ast } Exposur{e}_j\kern0.5em +\kern0.5em {\beta_i^c}^{\ast } Cluste{r}_j $$

Patient contrasts were coded p1_vs_p3 and p1_vs_p2, exposure contrasts coded hiv_vs_media, and cluster contrasts codes c2/3/4/5_vs_c1. Two-sided t-tests identified 131 and 14 genes that were significantly associated with patient and exposure, respectively (Bonferroni-adjusted *p* value < 0.01), while 1170 genes were significantly associated with cluster contrasts. These numbers suggest that cluster identity is far more determinant of global gene expression than patient or exposure. Cluster proportions are themselves associated with patient and exposure condition: for c1/2/3/4, we modeled the relative abundance of cluster k as a logistic model of Patient and Exposure:

$$ P\left({c}_k\right)\sim {\alpha}_k+{\beta}^{p\ast } Patient+{\beta}^{e\ast } Exposure $$

While all four clusters exhibited significant association by patient (*p* < 0.05), all but c2 showed significant evidence (*p* < 0.05) of exposure dependence: the c1 proportion was enriched by HIV exposure, while both c3 and c4 were depleted by the exposure.

In patients p2 and p3, for which viability sorts were applied to some batches, we observed similar cluster compositions across both exposure conditions at 48 h (Additional file 1: Figure S2I), suggesting that pre-selection of viable cells does not affect the distribution of the clusters identified and analyzed in this study. Instead, the effect of viability sorting appears to be the depletion of a large, low-quality cluster exhibiting low B2M expression uncharacteristic of mDCs (Additional file 1: Figure S2J, K).

Twenty-four-hour samples were assigned partial cluster identities by projecting them into the first seven PCs of the 48-h data. Following projection, the 30 nearest 48-h neighbors (Euclidean distance) were identified and used to assign partial memberships proportional to the memberships of the neighbors (Additional file 1: Figure S3B, C).

### Reproducible module gene analysis

scRNA-seq is a powerful technique that can identify functionally important subgroups of cells and their complex underlying biology. As scRNA-seq has become a more mainstream technology, new questions about how to model single-cell variation have continued to arise. To date, applied computational modeling approaches have mostly described single-cell heterogeneity as a combination of gene-intrinsic effects (i.e. fundamental molecular noise) [51] and gene-extrinsic ones, with the latter capturing both cell-intrinsic features (e.g. differences in intracellular protein levels, epigenetic state, mutation status, extracellular environment) and library-intrinsic technical artifacts (e.g. drop-out effects) [52, 53]. Yet, in single-cell studies that utilize samples from across multiple donors (e.g. patients or mice), these gene-extrinsic sources can be further subdivided into those that are unique to specific donors and those that are shared. The category of donor-dependent variation ranges from donor-specific cell subsets and large differences in cell-type composition to more subtle expression differences in constituent cell types. If the goal of a study is to generate hypotheses relating to a common phenotype, strategies for prioritizing shared features can benefit from quantitative characterizations of reproducibility across multiple donors.

Our clustering analysis captured the full distribution of cell states seen across the three ECs, but we also attempted to identify clusters of genes—gene modules—that were consistently co-regulated across patients at 48 h. Unlike DE analysis, this unsupervised approach aims to identify transcripts serving as reliable proxies of reproducible gene expression patterns.

We first pooled the normalized log-TPM data for each patient and separately computed the gene–gene Pearson correlation matrix. Each correlation value was Fisher-transformed and scaled to a z-score with 0 median and a median absolute deviation (MAD) equal to 0.67 (computed over the upper triangle). Only gene pairs with abs(z) > 2.4 in all three patient matrices were considered “reproducible gene pairs.” This step is implemented in the *scRAD::get.repro.thresh.adjacency* function in R.

For each gene, we tallied the number of reproducible gene pairs to which it belongs. We considered whether we could find genes with significantly more pairs than would be expected by chance; these genes could serve as reliable proxies of reproducible correlations. The distribution of pair counts was modeled as a zero-inflated Poisson process, including a randomly connected Poisson component and an unconnected zero-component. Under this null model, we computed upper-tail *p* values using the *scRAD::pzipdegree* function, identifying 263 genes with *p* values < 0.01 after Bonferroni adjustment. As these genes are connected to a large number of reproducible gene pairs, we called these proxy genes “reproducible module genes.”

Complete clustering of the median gene-gene correlation across the three patients (using correlation distance) demonstrates how these genes cluster into three specific modules (Additional file 1: Figure S6A, B).

### Single-cell signature analysis

We searched Gene Expression Omnibus (GEO; https://www.ncbi.nlm.nih.gov/geo/) for all study entries matching the query: “((“homo sapiens”[Organism] NOT “mus musculus”[Organism]) AND (“expression profiling by array”[DataSet Type] OR “expression profiling by high throughput sequencing”[DataSet Type])) AND (“dendritic cell”[Sample Source] or “dendritic cells”[Sample Source])”, utilizing the results to identify relevant expression signatures from the MSigDB C7 collection. We then applied FastProject^10^ to identify representative expression signatures in our normalized TPM data. Signature inputs include the selected MSigDB signatures, a curated signature of 28 IFN-response genes [20, 23], three unsigned signatures of our reproducible modules, and a pre-computed cluster signature. Results show that PCs 1 and 3 represent both biological signatures and reproducible module signatures more faithfully than alternative 2D projections (including the tSNE plot from Fig. 1B; Additional file 3: Table S2). In this reproducible “consensus” space, c3, c4, and c5 lie close together, while c1 and c2 are still well distinguished (Additional file 1: Figure S3A).

We selected a few of the top signatures from our FastProject analysis, considering the cumulative distribution of signatures across each of the five clusters (Fig. 2a, b, Additional file 1: Figure S5A). Two-sided KS tests were performed between the signature distributions of c1 (*n* = 20), c2 (*n* = 26), and c3 (*n* = 35) in order to monitor the extent to which these signatures discriminate the populations.

### DE analysis

Based on our signature analysis above, we considered three DE comparisons: (1) c1 vs c3, c4, and c5 (or “c1 vs c3-5”); (2) c2 vs c3, c4, and c5 (or “c2 vs c3–5”); and (3) c1 vs c2. Any DE analysis downstream of de novo clustering analysis demands careful consideration. Traditional DE analysis aims at identifying transcripts that vary markedly by sample class; a common goal is to rank the relative importance of transcripts in characterizing underlying expression states. Within the single-cell context, cell class is frequently defined based on low-dimensional representations of expression data. Therefore, the assumption that most genes are not differentially expressed between classes may not hold. Null models based on this assumption are ill-suited to the data, and will naturally yield uncalibrated probabilistic-based scores, e.g. deflated *p* value distributions.

In addition to biological factors, library intrinsic technical factors and batch-level features can drive broad expression covariance in scRNA-seq data. While some of these effects are random, others can confound DE analyses by systematically distorting transcriptome-wide differences between biologically distinct cell states. Without sufficient modeling efforts, batch specific biases can skew cluster classifications and reorder the ranks of DE genes.

A natural way to calibrate DE scores and monitor batch-specific effects is to consider measures of reproducibility over stratified, replicate experiments: in our case, over multiple patients. Unfortunately, there is no natural analog for biological replicates in the single-cell context; we do not yet wield the necessary experimental controls to reproduce a specific sample of transcriptional states. At the very least, we can map clusters from replicate experiments so that cluster contrasts are made comparable. For example, in our analysis we clustered cells from all patients simultaneously - offering a natural mapping between clusters called in the three patients: e.g. c1 cells in patient 1 belongs to the same biological “pseudo-replicate” as c1 cells in patient 2.

Our meta-analytical DE approach, implemented using the *scRAD::kruskalIDRm* tool, relies on the reproducibility metric known as IDR [24]. This metric evaluates a matched set of “signals” measured in two or more replicate experiments. In this analysis, we pooled cells from patients 1 and 2 to define the first study stratum, and considered all cells from patient 3 to be the second replicate stratum. We pooled cells from patients 1 and 2 together because the fewest high-quality cells were sequenced in these patients; pooling them together increased average stratum power. Though we performed a two-replicate analysis, our scRAD package modifies the Expectation–Maximization (EM) algorithm from the *idr* CRAN package to handle three or more replicates (see *scRAD* vignette). Equations relevant to this extension can be found in the Additional Online Materials (Additional file 7: AOM).

We performed simple DE analysis in each replicate study using Kruskal–Wallis tests; for each comparison, this yielded two lists of log-fold-changes and two lists of *p* values (see Additional file 4: Table S3). The two-component IDR mixture model was used to fit the joint distribution of *p* values obtained from these tests. For each gene, we can estimate a probability that the gene is a member of an “irreproducible component” for which *p* values are high and uncorrelated vs a “reproducible component” for which *p* values are low and correlated. Sorting genes by increasing probability of irreproducibility, one can compute the cumulative probability of membership for all genes of same or lower rank, defining an IDR. Genes called with an IDR < 0.01 were reported as “differentially expressed.”

We compared this approach to DE effects estimated according to a more traditional model of log-expression in gene i in cell j:

$$ {g}_{ij}\sim {\alpha}_i+{\beta_i^p}^{\ast } Patien{t}_j+{\beta_i^c}^{\ast } Cluster\_ contras{t}_j $$

Additional file 1: Figure S6D shows a comparison of IDR values to Bonferroni-adjusted *p* values obtained from t-tests on ${\beta}_i^c$ within the context of a c1 vs c3–5 contrast. Our criterion is generally more conservative than the regression approach above, with many significant genes having high (poor) IDR values. The genes that meet the significance criterion but not the reproducibility one may be good candidates for patient-specific DE. In Additional file 1: Figure S6E, we show the first two PCs computed over the two significant gene subsets: high IDR and low IDR. These plots appear to show tighter clustering among patient-specific structures for the high IDR set. To test this effect, we binned genes from both sets according to their rounded log-expression across the dataset (excluding c2). By sampling bins evenly for both sets—1000 times—we saw that the average silhouette width for patient and cluster contrast (c1 vs c3–5) shifted. Silhouette widths were computed on Euclidean metrics over the top one-third of PC space. Patient clustering was tighter for high IDR genes, while cluster contrasts were tighter for lower IDR genes. These results exemplify how our meta-analysis approach targets covariance structures shared across patients.

If we assume the difference between c1 and c2 is small compared to their common differences with clusters c3, c4, and c5 (c3–5), we may claim that the more a gene is reproducibly differentially expressed for one comparison, the more likely that gene should be reproducibly differentially expressed in the other. By this assumption, IDR analysis can be applied to two lists of IDR values from separate experiments in order to identify genes for which IDRs obtained from both comparisons are themselves correlated vs uncorrelated. Genes passing this threshold and showing common sign of DE were called “Shared” genes in Fig. 2c. Some of the remaining differentially expressed genes from these two comparisons were partitioned into three additional groups: (1) “c1-specific,” for which a gene is called differentially expressed in both c1 vs c3–5 AND c1 vs c2 comparisons, but not c2 vs c3–5; (2) “c2-specific,” which is analogously defined; and (3) “discordant,” for which genes are called differentially expressed in all three comparisons.

Given the large number of c1 cells at 48 h, we additionally considered the expression modulating effects of viral exposure vs media exposure to cells from a single cluster. Cells from p1 and p2 were pooled for a similar IDR analysis, though given the small number of cells tested (Additional file 4: Table S3) we imposed an additional reporting criterion of twofold difference to call genes as “differentially expressed” (Additional file 1: Figure S4C).

Candidate surface markers for c1 were identified using the *scRAD::getMarkers* tool. This tool reports the intersection of three gene sets: (1) genes differentially expressed between c1 and c3–5; (2) reproducible module genes; and (3) predicted membrane molecules from the Human Protein Atlas (http://www.proteinatlas.org) (Fig. 3a).

### Ingenuity pathway analysis

For each of the main three DE comparisons, we applied IPA [11] (https://www.qiagenbioinformatics.com) to the list of log-fold-change (mean of log-fold-changes from two replicate tests) and IDR, setting a less restrictive cutoff of IDR < 0.05 (Additional file 6: Table S5). The user dataset was used as the reference set for *p* value calculation and all experimentally verified mammalian associations were included in the analysis. IPA reported Benjamini–Hochberg q-values for canonical pathways enrichments and we performed our own Bonferroni *p* value adjustment for all reported upstream analysis *p* values.

Q–Q plots comparing putative upstream regulator log-p-values from c1 vs c3–5 and c2 vs c3–5 analyses displayed (not-shown) evidence of biased *p* value inflation between the two lists that could be adjusted via linear regression, producing “Adjusted” c1 vs c3–5 overlap *p* values for visualizing the results of these tests (Fig. 2e). In this visualization, two special sets of regulators were identified: (1) “common” regulators that are shared by the c1 vs c3–5 and c2 vs c3–5 results; and (2) “polarized” regulators that are unique to the c1 vs c3–5 results, but also shared with the c1 vs c2 results.

### False-negative weights

Due to small starting amounts of RNA, scRNA-seq data are burdened by “drop-out effects,” in which an mRNA transcript expressed in a cell is not detected during sequencing [54]. Dropped transcripts could have been degraded before library preparation or skipped by reverse-transcription. The probability of drop-out is largely a function of transcript abundance at cell lysis and there have been multiple empirical observations that drop-out rates and log-transcript abundance typically follow a logistic relationship [52, 54] Here, we analyze transcript detection rates using the following model:

$$ {\displaystyle \begin{array}{l}P\left({E}_{ij}\kern0.5em =\kern0.5em 1\right)\kern0.5em =\kern0.5em {\alpha}_j\\ {}P\left({D}_{ij}\kern0.5em =\kern0.5em 1|{E}_{ij}\kern0.5em =\kern0.5em 1\right)\kern0.5em =\kern0.5em \frac{1}{1\kern0.5em +\kern0.5em \exp \kern0.5em \left(-{Z}_i{\mu}_j\kern0.5em +\kern0.5em {S}_i\right)}\end{array}} $$

where D is an observed n × J binary matrix encoding whether or not transcript j is detected in cell i: 1 = Detection, 0 = No detection. E is a hidden binary matrix of the same dimension encoding whether or not the transcript is expressed in the cell: 1 = Expression, 0 = No expression. α_j_ is the estimated expression rate (i.e. % cells that express the gene *j*), μ_j_ is the median transcript abundance (here, normalized TPM) across all cells expressing the gene *j* (TPM > 0), and Z and S are n-vectors of cell-specific drop-out coefficients. We first fit a GLM to data from 99 genes with the lowest Fano-factor, adjusting for mean expression, assuming that these genes are expressed in all cells; this fit yielded both Z and S in the second equation. We then estimated E and α_j_ using the EM algorithm implemented in the SCONE package. Using the resulting posterior E probabilities (the expected E matrix), we computed a “weight matrix,” W_ij_, capturing the posterior probability that gene *j* did not drop out in sample *i*.

### Quantifying viral abundance

For each cell, viral abundance was quantified as a mean of RSEM TPM estimates for Gag and Pol gene segments (GenBank accession AF324493), given even coverage observed across those segments (Additional file 1: Figure S4A, B). Interestingly, we only observed HIV coverage in samples from p1. We applied Fisher’s exact tests to compare HIV detection across virally exposed subpopulations (excluding p1-specific cluster 5), but found no significant trends. Similarly, Kruskal–Wallis tests comparing gene expression in HIV-1-positive and -negative groups (all exposed) found no significant intra-cluster variation.

### Validation of c1-enriched CD64^Hi^,PD-L1^Hi^ population

As with the scRNA-seq data, we applied RSEM alignment and sample-filtering procedures to population RNA-seq samples sorted by c1 candidate markers, leaving 13 samples covering eight possible conditions (24 h/48 h, HIV/media, Hi/Lo). Expression values for 6557 genes were normalized using standard DESeq scaling normalization [55], followed by gene-level regression on the first PC of QC metrics, retaining the residual for downstream analysis. Duplicate gene symbols were averaged as above. A total of 576 of the differentially expressed gene symbols from the c1 vs c3–5 comparison, passing TPM gene filter, were detected in population experiments. A weighted mean was computed for each of these shared genes, for each single-cell subpopulation c1–5, and Pearson correlations were computed between sorted populations and population means after log1p-transforming both datasets. Radar plot cycles representing these correlations are presented on a min-max scale per bulk condition (min: 0.32–0.54, max: 0.81–0.89). Correlation values for replicate sample conditions (*n* = 2) were averaged before plotting.

### Prediction of upstream regulators of c1

In order to generate hypotheses related to the sensing mechanisms behind c1 response, we performed IPA (as described above) and identified several innate immune pathways—included specific TLR signaling pathways—selectively induced in this population. Due to the limited availability of genome-wide human stimulation data, we opted to compare our single-cell expression profiles to publicly available expression profiles of mouse bone-marrow-derived dendritic cell (BMDC) populations exposed to five TLR agonists (lipopolysaccharide (LPS), Pam3CSK4 (PAM), Polyinosinic:polycytidylic acid (Poly I:C), gardiquimod (Gard), and CpG DNA (CpG)) and one control (unstimulated) condition [33]. Replicate microarray samples from each condition were averaged, followed by averaging over probes of a gene symbol. Homologs were mapped using the biomaRt Bioconductor package and only uniquely mapping genes were considered for further analysis. Normalized single-cell TPM was log1p-transformed and gene abundances were centered by weighted means (using the W false-negative weight matrix defined above). Mouse data was log-transformed and genes were centered by their mean value. We computed a weighted correlation estimate (using the W matrix) for each pair of single-cell and mouse population taken at the 24-h time-point of the mouse study. For each bulk sample, we applied two-tailed Wilcoxon rank-sum tests to examine differences in correlation between cells from c1 and c3-5. The correlations were referred to as the “TLR stimulation score,” as they measure the extent to which the subpopulation-specific response is correlated with the TLR-stimulated profile. Using Stouffer’s z-method, we combined *p* values collected from the two donor pools used in DE (all implemented in *scRAD::kruskalMeta*) reporting a meta-analysis FDR < 0.01 (Fig. 4b) [33]. Weighted correlation of samples i and i' is defined by the equations:

$$ {\displaystyle \begin{array}{l}\mathrm{wMea}{\mathrm{n}}_i\kern0.5em \left(X,\kern0.5em W\right)\kern0.5em =\kern0.5em \frac{\varSigma_j{X}_{ij}{W}_{ij}}{\varSigma_{j\hbox{'}}\kern0.5em {W}_{ij\hbox{'}}}\\ {}\mathrm{wCo}{\mathrm{v}}_{ii\hbox{'}}\kern0.5em \left(X,\kern0.5em W\right)\kern0.5em =\kern0.5em \frac{\varSigma_j\kern0.5em {W}_{ij}\kern0.5em {W}_{i\hbox{'}j}\kern0.5em \left({X}_{ij}\kern0.5em -\kern0.5em \mathrm{wMea}{\mathrm{n}}_i\kern0.5em \left(X,\kern0.5em W\right)\right)\kern0.5em \left({X}_{i\hbox{'}j}-\mathrm{wMea}{\mathrm{n}}_{i\hbox{'}}\kern0.5em \left(X,\kern0.5em W\right)\right)}{\varSigma_{i\hbox{'}}\kern0.5em {W}_{ij\hbox{'}}\kern0.5em {W}_{i\hbox{'}j\hbox{'}}}\\ {}\mathrm{wCo}{\mathrm{r}}_{ii\hbox{'}}\kern0.5em \left(X,\kern0.5em W\right)\kern0.5em =\kern0.5em \frac{\mathrm{wCo}{\mathrm{v}}_{ii\hbox{'}}\kern0.5em \left(X,\kern0.5em W\right)}{\sqrt{\mathrm{wCo}{\mathrm{v}}_{ii}\kern0.5em \left(X,\kern0.5em W\right)\kern0.5em \mathrm{wCo}{\mathrm{v}}_{i\hbox{'}i\hbox{'}}\kern0.5em \left(X,\kern0.5em W\right)}}\end{array}} $$

Where weights of population data are set to unity.

We next sought to generate analogous results using RNA-seq data collected from human mDCs rather than distant mouse BMDCs. We applied RSEM alignment and sample-filtering procedures to population RNA-seq data collected from DCs incubated for 48 h with or without various TLR ligands (see above), leaving eight samples covering five possible conditions (no TLR, TLR2/3/4/8). Expression values for 18,482 genes were normalized using standard DESeq scaling normalization [55]. Duplicate gene symbols were averaged as above. We applyied the same meta-analysis pipeline as for the mouse array data, ranking various inductions in their relative similarity to c1 (Additional file 1: Figure S10A).

Drawing again on available characterizations of the mouse BMDC system, we chose to correlate single-cell gene expression profiles with short hairpin RNA (shRNA) knockdowns of TLR signaling network components) [32] to highlight potential upstream regulators mediating c1 response. Publicly available—and normalized—nCounter population data were mapped to unique human homologs, log-scaled and gene-centered as above. Normalized single-cell TPM estimates were similarly log1p-transformed and centered by weighted mean. Weighted correlation estimates were computed as in the TLR analysis above, and for each shRNA experiment, we applied two-tailed Wilcoxon rank-sum tests to examine differences in correlation between c1 and c3–5. The opposite of the correlation was referred to as the upstream regulatory score, as it measures the extent to which the sub- specific response is anti-correlated with the shRNA-knockdown profile. Instead of simple meta-analysis on the donor pools used for the TLR stimulation scores, we applied the *scRAD::kruskalIDRm* analysis as in the DE analysis, defining IDR < 0.05 as our threshold for calling differential signatures (Fig. 4f).

## Additional files

Additional file 1: Supplementary **Figures S1**–**S10**. (PDF 31607 kb)

Additional file 2: Table S1. Clusters and tSNE coordinates for 48-h cells. Cluster identifiers and tSNE coordinates from (Fig. 1c) as described in “Methods.” (XLSX 27 kb)

Additional file 3: Table S2. Reproducible Modules and FastProject Analysis. Lists of genes exhibiting significantly high numbers of reproducible correlations as described in “Methods.” FastProject signature values and significance scores. (XLSX 408 kb)

Additional file 4: Table S3. DE Analysis. DE analysis results as described in “Methods,” including contrasts for c1 vs c3–5, c2 vs c3–5, c1 vs c2, and intra-c1 exposure differences. Numbers of cells in each comparison are printed above log-fold-change columns. Gene sets from Fig. 2c are included. (XLSX 3450 kb)

Additional file 5: Table S4. Patient metadata and biomarker data. Clinical data summaries for patient groups and anonymized biomarker values for elite controllers and chronic progressors: CD4^+^ T cell counts, viral load, and CD64^Hi^,PD-L1^Hi^ fractions before and after viral (VSV-g pseudotyped HIV-1) exposure. (XLSX 39 kb)

Additional file 6: Table S5. IPA. Canonical pathways and upstream analysis for DE results: contrasts for c1 vs c3–5, c2 vs c3–5, c1 vs c2. (XLSX 203 kb)

Additional file 7: AOM. Additional online materials. (PDF 243 kb)
