# Supplementary material for: A Reproducibility-Based Computational Framework Identifies an Inducible, Enhanced Antiviral State in Dendritic Cells from HIV-1 Elite Controllers
Source: Genome Biol. 2018 Jan 29;19:10. doi: 10.1186/s13059-017-1385-x (PMC5789701; doi:10.1186/s13059-017-1385-x)
Supplement: Supplementary file 1 — Supplementary Figures S1–S10. (PDF 31607 kb) [file 13059_2017_1385_MOESM1_ESM.pdf]

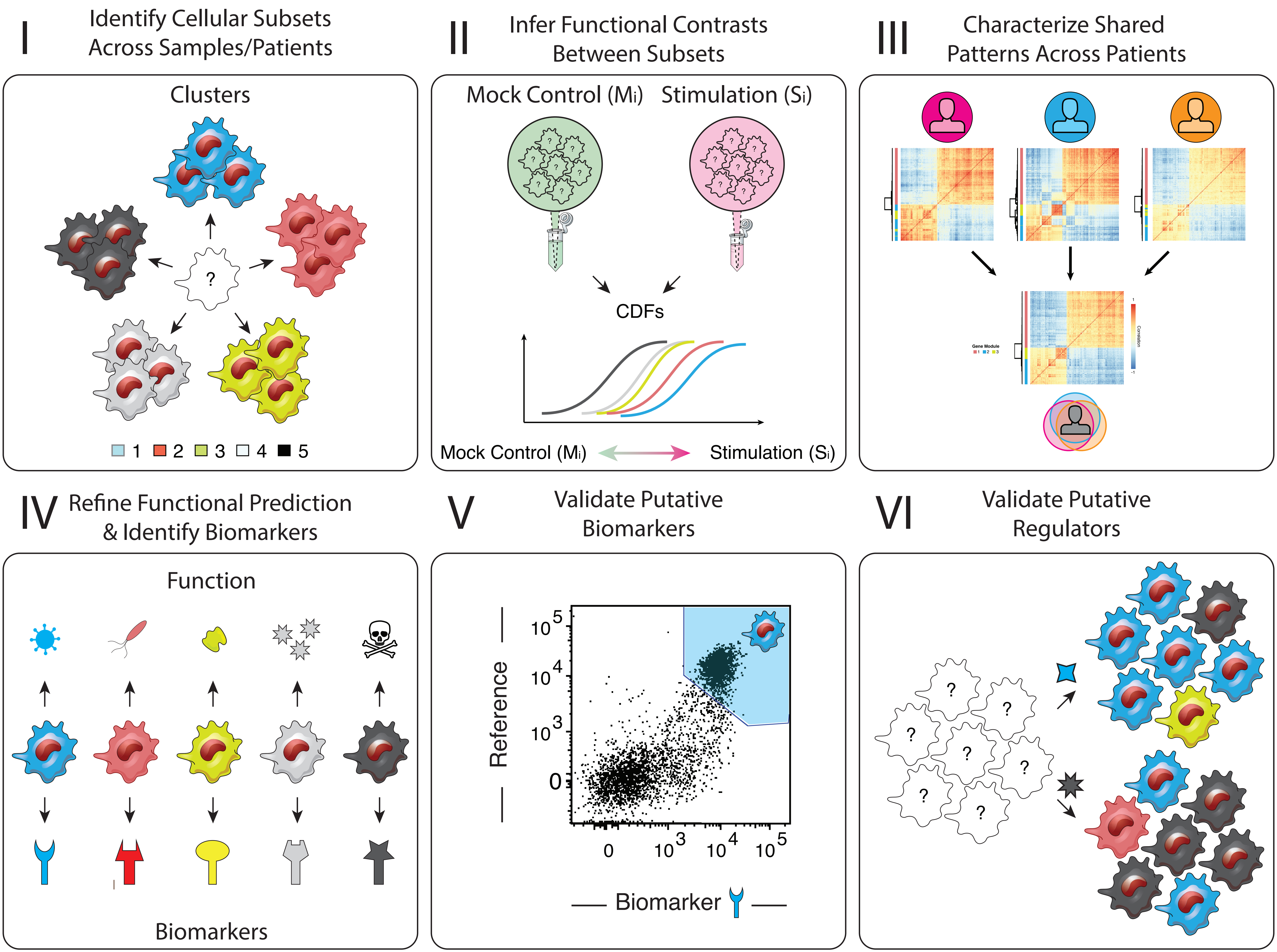

Additional Figure 1 I A rational framework used to resolve, characterize and then modulate response states across multiple patient sources. Our generally applicable framework is founded upon the following workflow: (I) Resolve the individual mDC subtypes and states that comprise the system under study (Methods); (II) Define putative functions for each and identify biologically meaningful contrasts using existing databases; (III) Characterize patterns of differential expression that are common across patients; (IV) nominate potential biomarkers and relevant cellular circuitry based on accumulated knowledge; (V) Isolate and characterize interesting subsets; (VI) validate inferred regulators.

Additional Figure 2

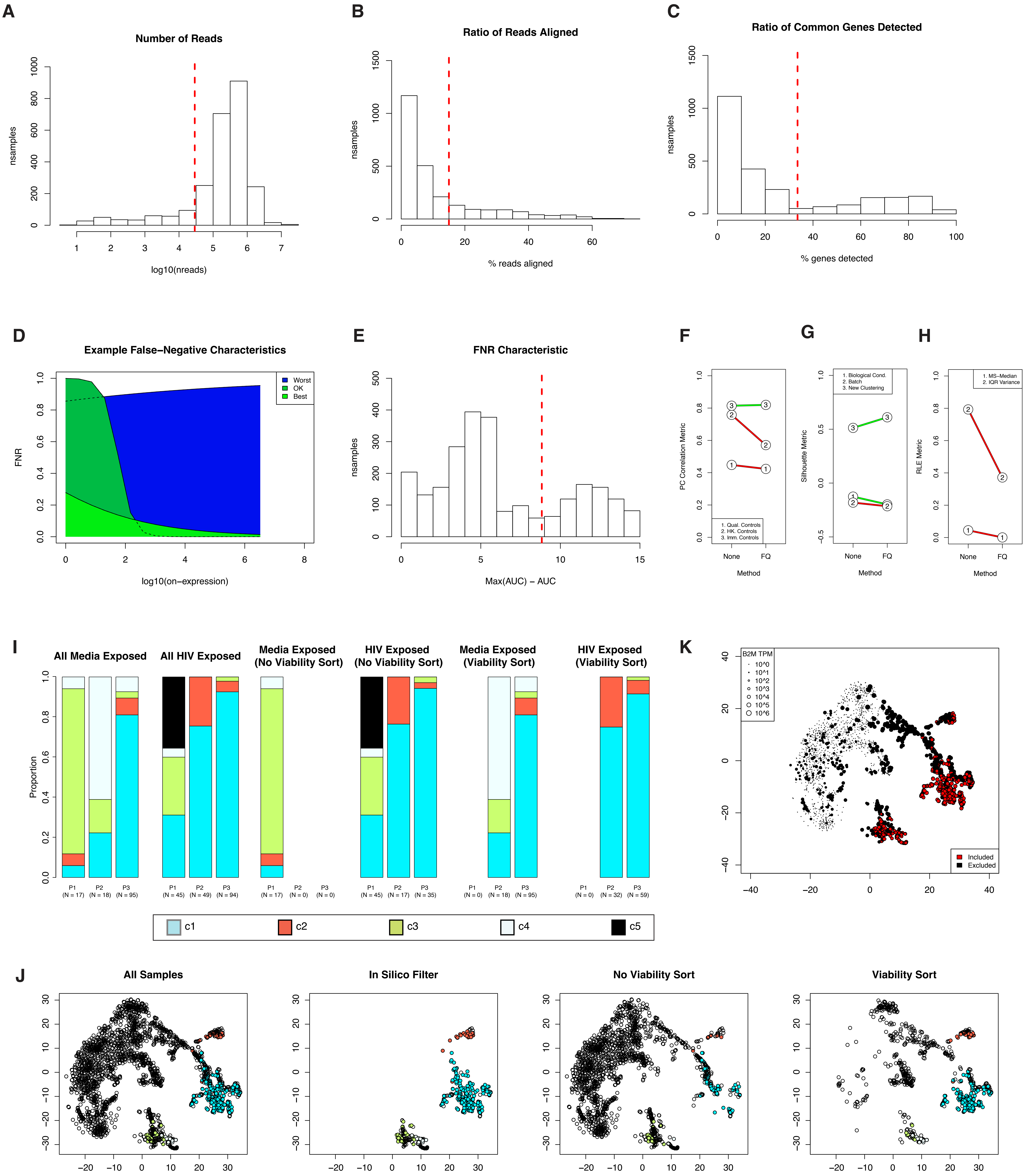

Additional Figure 2 I Single-Cell Filtering and Normalization. (A-C, E) Distributions of single-cell sample (24 and 48 hours) filtering metrics. Red lines represent adaptive threshold below which all samples (n = 2,489) were removed from further analysis (see SOM). (A) Distribution of number of paired-end reads per library. (B) Distribution of transcriptome read alignment ratio per library. (C) Distribution of the fraction of "common genes" detected per library. (D) Example false-negative characteristic fits, exhibiting three different relationships between the mean on-expression (TPM) of constitutively expressed genes and their false negative rate (FNR) or "drop-out rate." Colors represent the overall quality of the area under the curve (AUC) (see SOM). (E) Distribution of fit FNR AUC per library. (F-H) Differences in SCONE metrics before and after full-quantile (FQ) normalization (see Methods). (F) Correlation between the first 3 expression Principal Components (PCs) and the first 3 PCs computed across "negative" controls (Alignment QC Metrics and housekeeping (HK) genes) tend to decrease while correlations with the first 3 PCs across "positive" controls (innate immune system genes) tends to increase. (G) The average silhouette width (ASW) of biological condition (patient x exposure x timepoint x viability sort) and the ASW of batch both decrease. However, the ASW of de-novo clustering tends to increase. (H) The mean sample-median relative log-expression (RLE) decreases, as does the variance of the sample inter-quartile range RLE decrease: both global differential expression and differential expression variability is reduced. (I) Stacked bar plots depicting the percentage of total mDCs in each 48h cluster c1-5 (see AOM) for each patient under media and viral exposure conditions, stratifying by no viability or viability pre-selection. Both types of single-cell libraries were only obtained from patients p2 and p3. As seen in HIV-1-exposed samples, viability-sorted compositions are comparable to samples without viability sorting. (J) tSNE plot of (un-normalized) log(TPM+1) expression, including all cells from 24 hours and 48 hours, HIV and Media exposures, with or without viability gating. Points are colored according to a 48h cell's membership to clusters c1-5. Various subsets are plotted independently, including cells passing in silico cell filter, cells that were not sorted on viability, and cells passing viability sorting. Viability sorting tends to exclude cells from low-quality clusters, enriching the fraction of cells passing quality filtering. (K) tSNE plot from (J), sizing points according to estimated expression levels of B2M. Red samples passed in silico cell filter. Clusters of cells excluded by filter exhibited very low-levels of the housekeeping transcript.

Additional Figure 3

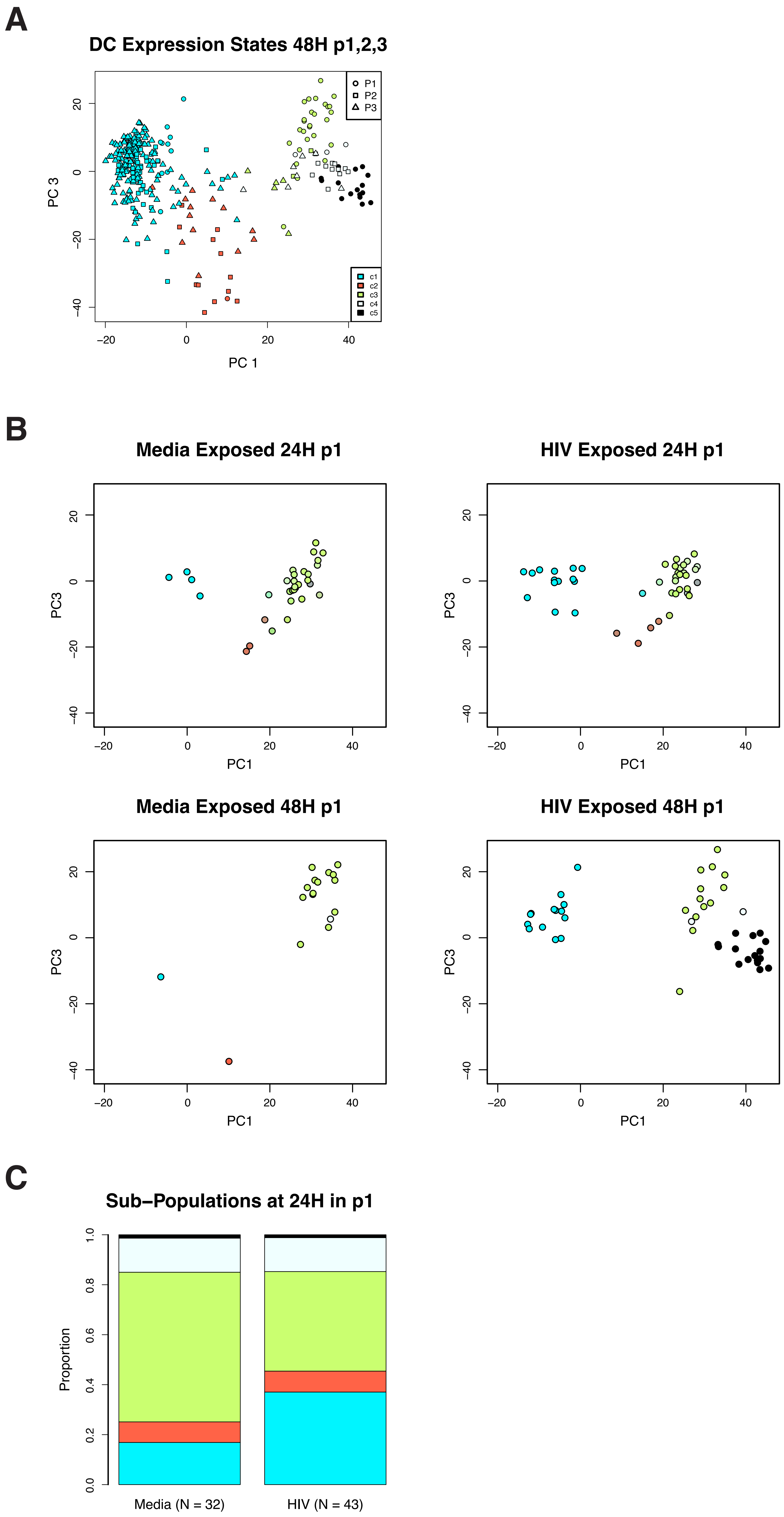

Additional Figure 3 I Single cell data structure at 24 hours in patient 1. (A) PCA of scRNA-Seq data from all three ECs (p1, p2 and p3) after 48 hours of incubation (Figure 1A,B). PCs 1 and 3 were selected by FastProject analysis (see AOM). (B,C) 24 hour samples were assigned fractional cluster memberships proportional to the 30 nearest 48 hour neighbors in the first 7 PCs (see AOM). (B) PCA representing mDCs from p1 incubated for 24 (top) and 48 (bottom) hours in media (left) or VSV-G pseudotyped HIV-1 (right), plotted in the same PCA space as in (A). Data points for 24 hour samples are assigned the average color of their nearest 48 hour neighbors. (C) Stacked bar plot depicting expected percentage of total mDCs in each cluster for p1 at 24 hours under media and viral exposure conditions.

Additional Figure 4

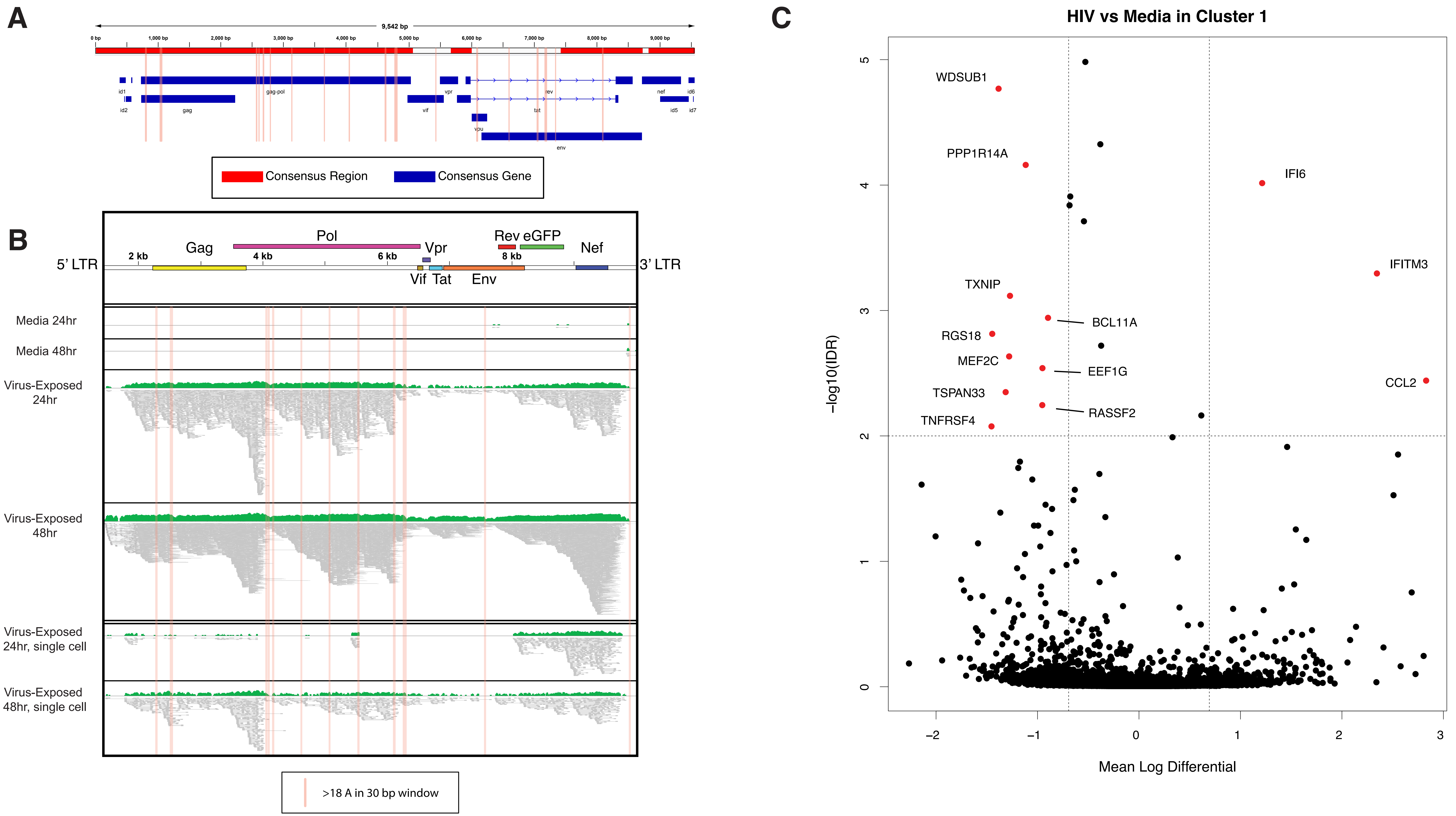

Additional Figure 4 I Impact of HIV on c1 and viral expression in single cells. (A,B) Intracellular viral products can be captured with scRNA-Seq. (A) Top: HIV-1 B consensus genome (http://www.hiv.lanl.gov/) with regions homologous to pseudotyped virus highlighted in red (BLAST). Bottom: consensus genes shown in blue. Adenine-rich regions (> 18 A in 30 bp window) could potentially anneal primers used in SMART-Seq2 and are marked with vertical bars. (B) Alignment of reads (reads in grey; histogram of reads in green) from pooled media or virus-exposed p1 cells at 24 and 48 hours (top) to the viral sequence between the 5'LTR and 3'LTR of our pseudo-typed viral plasmid (bar at top, colored by gene). Representative single cells are shown at bottom. Vertical bars mark positions in the plasmid sequence where there are at least 18 Adenines in a 30-base pair window. (C) Identification of genes enriched in VSV-G pseudotyped HIV-1 vs media exposure conditions across cells from c1: negative log-10 (Irreproducible Discovery Rate) is plotted against mean log-fold differential expression (see Methods). Genes differentially up-regulated in HIV (right) or media (left) are highlighted in red and labeled.

Additional Figure 5

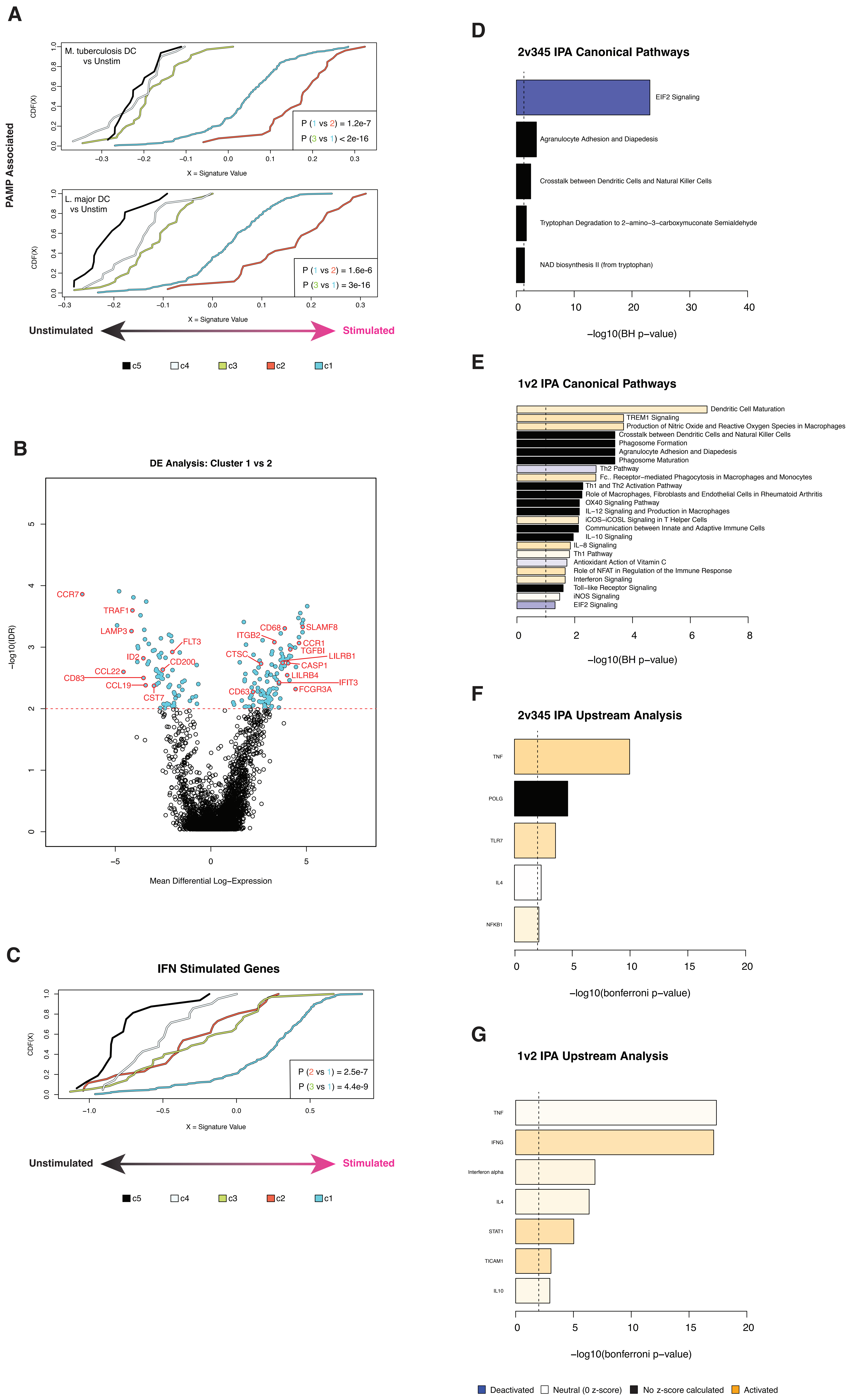

Additional Figure 5 l Analysis of immune activation across clusters. (A) Cumulative distribution function (CDF) comparisons for single cells from each cluster identified in Figure 1B with FastProject gene signatures derived from the GSE360 GEO accession (see AOM). The single-cell signature value quantifies the extent to which each cell is polarized toward stimulated vs unstimulated expression states. Clusters with gene expression signatures more closely mapping to the stimulated condition shift right, while clusters characteristic of un-stimulated shift left. Kolmogorov–Smirnov (KS) tests show significant differences in these signatures between the first 3 clusters (c1, n = 220; c2, n = 26; c3, n = 35). (B) Volcano plot of negative log irreproducible discovery rate (IDR) vs mean differential log-expression between clusters 1 and 2 (c1 and c2) (see AOM). Selected genes are labeled in red. (C) CDF plot for an unsigned FastProject signature of (n = 28) interferon stimulated genes. As in (A), clusters with stronger interferon (IFN) stimulated gene signatures are shifted right. KS tests show c1 has a significantly higher IFN signature than c2 or c3. (D,E) Selected ingenuity Pathway Analysis (IPA) (see AOM) results (Benjamini-Hochberg q-value < 0.01) for enriched Canonical Pathways significantly deactivated (blue), neutral (white: with z score; black: without z score) or activated (orange) in (D) c2 vs combined c3-5 and in (E) c1 vs c2. (F,G) Selected IPA Upstream Analysis results (Bonferroni p-value < 0.05) for (F) c2 vs combined c3-5 and for (G) c1 vs c2.

Additional Figure 6

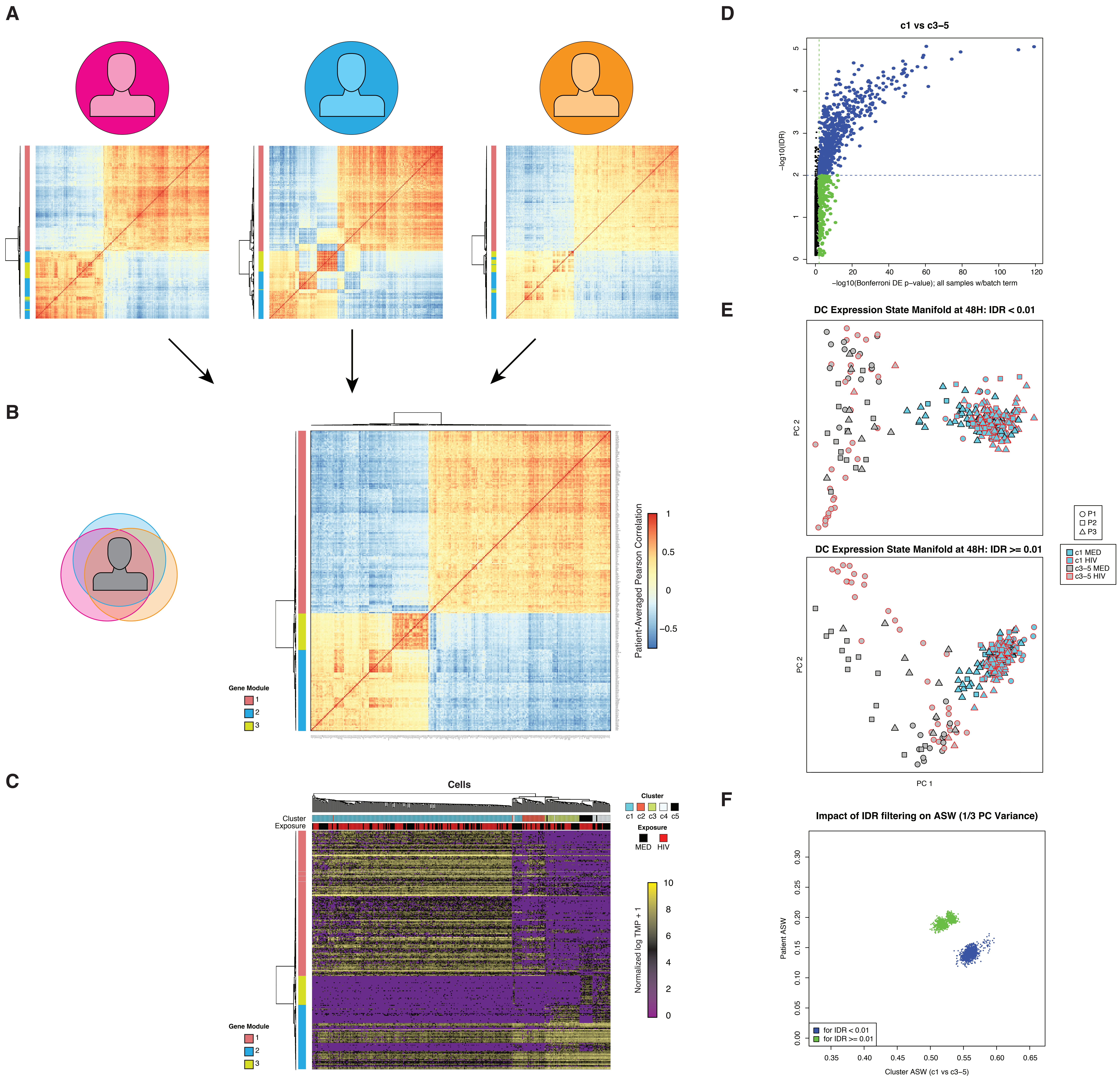

Additional Figure 6. Reproducible gene modules resolve cluster responses. | (A,B) Hierarchical clustering of the median (across 3 patients) gene-gene correlation matrix for genes reproducibly expressed across all three EC patients (see Methods). Genes are clustered by complete-linkage clustering on correlation distance. Reproducible module genes may be clustered into three modules (m1-m3). (C) Expression heatmap showing hierarchical clustering of genes (from reproducible modules in A) by cells (48 hour single cell data from all 3 ECs - p1, p2, p3). Cells are clustered by the UPGMA method on Euclidean distances. (D) For each gene, comparison of IDR differential expression criterion (y-axis) to traditional regression analysis p-value criterion (x-axis), the latter adjusting for batch (patient) effect. Blue genes meet both criteria, while green genes meet only the traditional criterion; IDR selection is generally more conservative than the alternative. (E) PCA plots showing the clustering by patient and sub-populations for (top) traditionally significant and reproducible genes with IDR < 0.01 and (bottom) significant but irreproducible genes with IDR > 0.01 (F) Each point corresponds to a sub-sampled PCA analysis as in (E). High IDR (blue) and low IDR (green) genes from (D) are sub-sampled 1000 times to maintain comparable expression means across sets (see Methods). Euclidean cell-distance metrics are computed over each set, filtering expression data to the top 1/3 of PC variance. Average silhouette widths (ASWs) are computed for patient condition (y-axis) and cluster condition (x-axis); while patient effects decline upon IDR selection, cluster contrasts improve.

Additional Figure 7

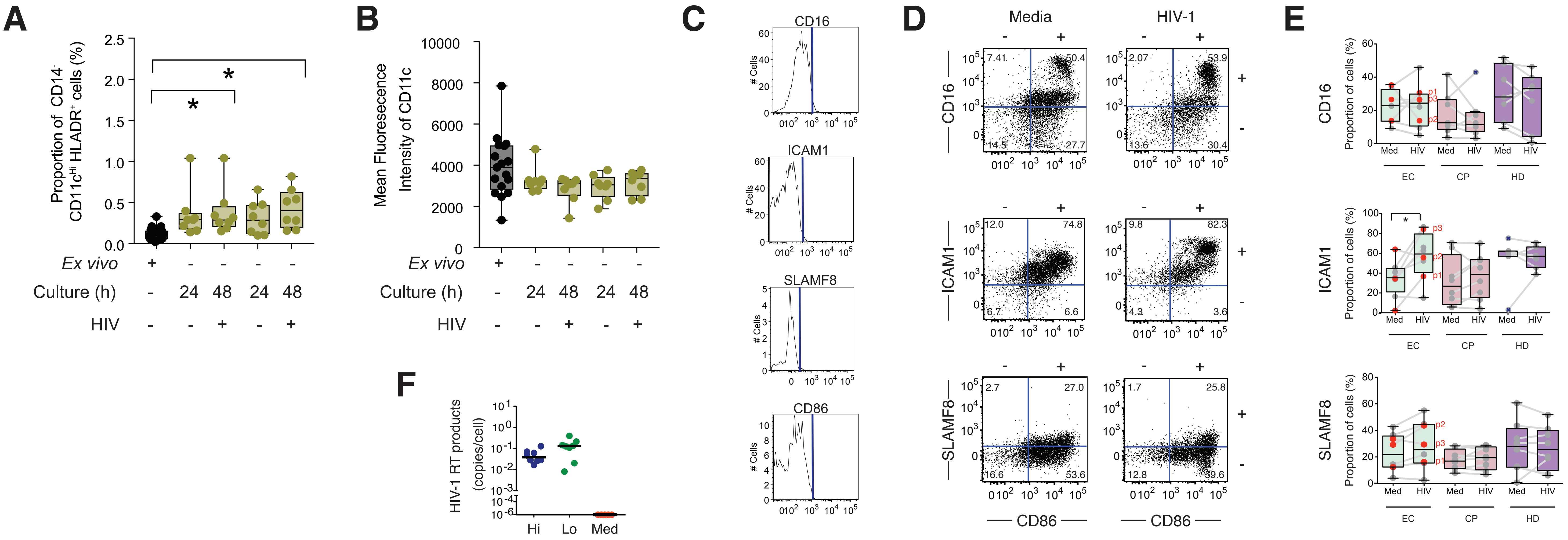

Additional Figure 7 I Testing of membrane marker candidates for c1-like mDCs and quantification of HIV-1 reverse transcripts. Analysis of the frequency of CD14- CD11cHi DR+ mDC (A) and Mean Fluoresence Instensity (MFI) of CD11c in mDC (B) present in PBMC from elite controllers (ECs) ex vivo (black, n=17) or cultured in vitro for 24 and 48h in the presence or either media alone (Med) or VSV-G-pseudotyped HIV-1 (green, n=8). Significance in frequency of CD14- CD11cHi HLADR+ mDC between ex vivo samples and 24 and 48h HIV stimulation was found using a One ANOVA test and a Tukey's multiple comparisons test (*; adjusted p value= 0.0409 and 0.0105, respectively). No significant differences were observed for CD11c MFI using the same test (B). (C) Representative flow cytometry analysis of CD86 (x-axis) versus either CD16 or ICAM1 or SLAMF8 (y-axis) in gated CD14- CD11cHi HLADR+ DCs from EC p1 cultured for 24h in the presence of media (left) or VSV-G pseudotyped HIV-1 (right). (D) Summary of proportions of CD86Hi DCs from media and virus-exposed EC (green; n = 8), chronic progressors (CP, pink; n = 8) and healthy donors (HD, blue; n = 7) co-expressing high levels of each of the markers analyzed in (B). Statistical significance was calculated using a two-tailed Wilcoxon matched pairs rank test (*, $p < 0.05$). (E) Quantification of HIV-1 reverse transcript amounts by qPCR present in mDCs exposed in vitro for 24h to a VSV-G pseudotyped HIV-1 virus and sorted on high (Hi; Blue; n = 8) and low (Lo; green; n = 8) expression levels of CD64 and PD-L1. As a control, mDCs treated with media alone were included in the analysis as a control in some experiments (Orange; n = 5). Data indicates values of HIV-1 gag DNA amplification relative to endogenous CCR5 levels present in each DNA sample.

Additional Figure 8

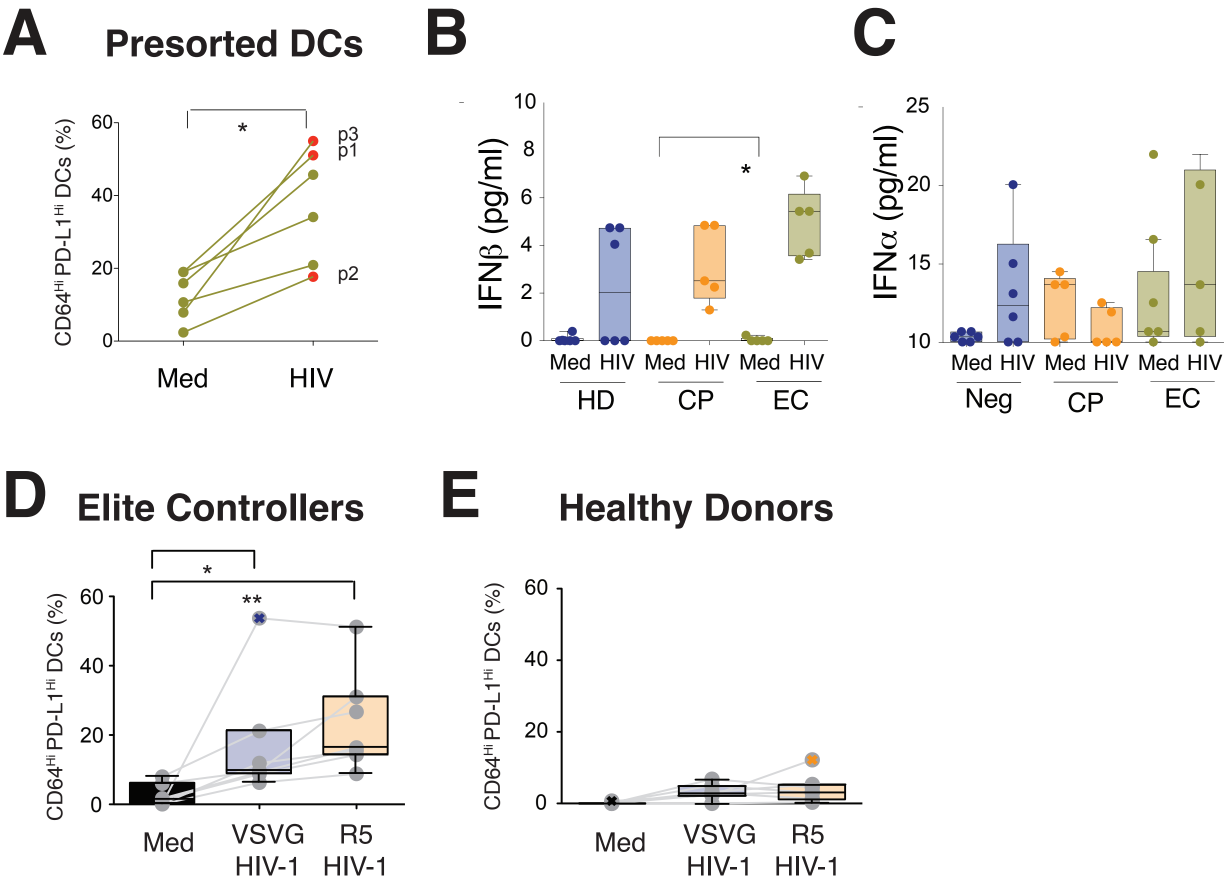

Additional Figure 8 I Induction of c1-enriched/CD64Hi,PD-L1Hi mDC in response to different HIV-1 strains and culture conditions. (A) Proportions of CD64Hi,PD-L1Hi cells detected in mDCs sorted prior to culture in the presence of media and VSV-G pseudotyped HIV-1 virus. Statistical significance was calculated using a two-tailed Wilcoxon test (*, $p < 0.05$; $n = 6$). (B) ELISA analysis of IFN beta protein levels present in culture supernatants of mDCs from healthy donors (Neg, blue; n=6), chronic progressors (CP, orange; n=5) and elite controllers (EC, green, n=5) exposed for 48h to either media (Med) or VSVGpseudotyped HIV-1 virus (HIV). mDCs were presorted from PBMC prior to in vitro culture. *p=0.0397, One tailed Mann Whitney test. (C) Luminex analysis of IFN alpha protein levels present in culture supernatants of mDCs from healthy donors (Neg, blue; n=6), chronic progressors (CP, orange; n=5) and elite controllers (EC, green, n=5) exposed for 48h to either media (Med) or VSVG-pseudotyped HIV-1 virus (HIV). Differences not significant. (D,E) Proportions of CD64Hi,PD-L1Hi cells included within CD14- CD11cHi HLADR+ mDC from elite controllers (D, n = 7) and HIV negative donors (E, n = 7) after 24h of culture in the presence of media or either VSV-G pseudotyped or CCR5 (R5) tropic HIV-1 virus. Statistical significance was calculated using a Kruskal-Wallis test followed by a Dunn's test (*, p = 0.0259; **, p = 0.0011).

Additional Figure 9

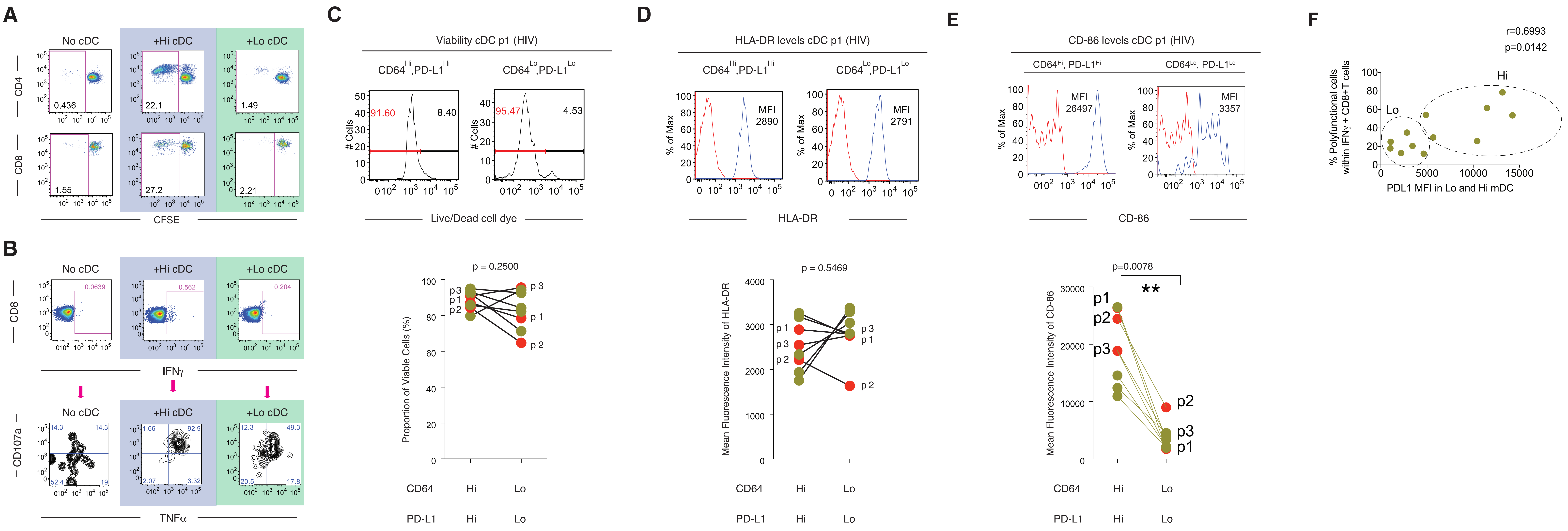

Additional Figure 9 I In vitro analysis of the functional properties of c1-like CD64Hi,PD-L1Hi mDC. (A) Representative flow cytometry analysis of CFSE levels present in CD4+ (upper panels) and CD8+ (lower panels) T cells from a healthy donor co-cultured in the absence (left) or presence of allogeneic mDC from EC patient 1 exposed to HIV-1 and expressing high (Hi, middle, blue) or low (Lo, right, green) levels of CD64 and PD-L1. Numbers on plots represent proportions of CFSElow proliferating T cells. Full data from n = 6 patients presented in Figure 3G. (B) Flow cytometry analysis of CD8 vs intracellular levels of IFN present in gated CD8+ T cells cultured in the absence (No DC, left) or the presence of autologous mDC exposed to HIV-1 and expressing either high (Hi DC, middle, blue) or low (Lo DC, right, green) levels of CD64 and PD-L1. Expression of CD107a and intracellular TNF was analyzed on gated IFN positive cells in each condition (lower panels). Numbers in quadrants represent proportions of positive cells. Full data from n = 7 presented in Figure 3H&J. (C,D) Analysis of viability and HLA-DR levels in mDCs subsets. (C): Representative flow cytometry analysis of cell viability by Live/Dead cell dye staining of mDCs from patient 1 expressing high or low levels of CD64 and PD-L1 24h after exposure to VSV-g pseudotyped HIV-1. Unstained cells (in red) represent viable cells. Summary of viability in different patients is shown in the lower panel (n=8). P-value calculated using a two-tailed Wilcoxon signed-rank test is shown above the plot. (D): Overlay representation of HLA-DR levels expressed by CD64Hi,PD-L1Hi and CD64Lo,PD-L1Lo mDCs (blue) induced after 24h of exposure to VSV-g pseudotyped HIV-1. Background staining levels are shown in red. A summary of HLA-DR levels from the mentioned CD64Hi,PD-L1Hi and CD64Lo,PD-L1Lo mDCs from n=8 patients is shown in the lower panel. P-value calculated using a two-tailed Wilcoxon signed-rank test is shown above the plot. (E) Analysis of Mean Fluorescence Intensity of CD86 in gated CD64Hi PD-L1Hi and CD64Lo PD-L1Lo subpopulations included in CD14- CD11cHi HLADR+ mDCs from controllers cultured for 24h in the presence of VSVG-HIV-1 virus. Representative histograms are shown in the upper panel. Original patients 1,2,3 (p1,p2,p3) used for sc-RNAseq analysis are highlighted in red. **; p= 0.0078, two tailed Wilcoxon matched-pairs signed rank test. (F) Spearman correlation of PD-L1 mean fluorescence intensity levels present in sorted CD64Hi PD-L1Hi and CD64Lo PD-1Lo mDC subpopulations induced after exposure to VSV-G pseudotyped HIV-1 and the corresponding proportions of polyfunctional TNF + CD107a+ CD8+ T cells included in IFN + autologous CD8+ T cells induced by these cells in functional assays. p value= 0.0142; Spearman coefficient r= 0.6993. Values corresponding to CD64Hi PDL1Hi (Hi) and CD64Lo PD-1Lo (Lo) subsets from n=6 EC with detectable CD8 T cell responses have been highlighted with circles.

Additional Figure 10

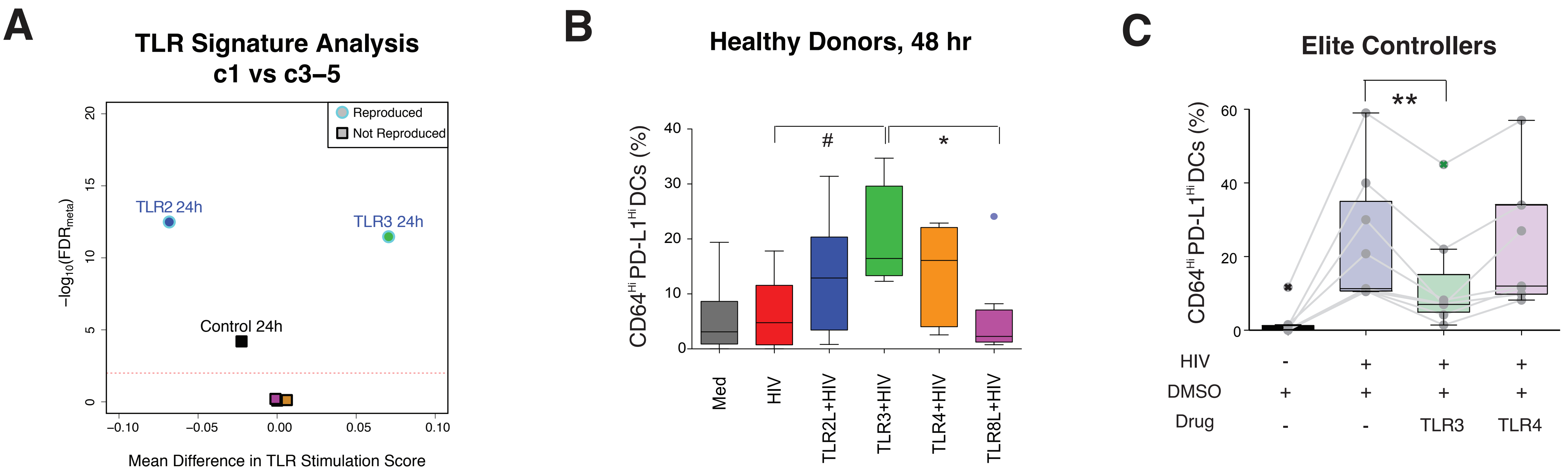

Additional Figure 10 I Impact of TLR ligands and inhibitors in the acquisition of the CD64Hi,PD-L1Hi phenotype. (A) Volcano plot of negative log meta-analysis false discovery rate (FDR) vs mean difference in "TLR stimulation score" between c1 and c3-5. Scores are computed from weighted correlations between single-cell profiles and transcriptional patterns from mouse DCs (Amit et al, Science 2009 (17); see AOM) after 24h of stimulation with media control (black) or agonists for either TLR2 (PAM3CSK4, dark blue), TLR3 (Poly I:C, green), TLR4 (LPS, orange), TLR7/8 (Gard, purple), or TLR9 (CpG, light blue). Tests reproduced with FDR < 0.01 in both stratified analyses are highlighted in blue. (B) Proportions of CD64Hi,PD-L1Hi cells induced within mDCs from healthy donors exposed to either media or VSV-G pseudotyped HIV-1 virus in the absence or the presence of the indicated TLR ligands (TLR2L, PGNA, n = 8; TLR3L, PolyI:C, n = 8; TLR4L, LPS, n = 5; TLR8L, CL097, n = 8) for 48h (24h data shown in Figure 4C). These data indicate that, even at later time points, TLR3 has the most significant impact on the acquisition of the CD64HiPD-L1Hi phenotype. Statistical significance was calculated using a two-tailed Wilcoxon signed-rank test (*, $p < 0.05$). (B) Proportions of CD64Hi,PD-L1Hi cells within gated mDCs (CD14-, CD11c+, HLADR+) from EC patients cultured in the presence or absence of VSV-G pseudotyped HIV-1 virus and either DMSO (control) or TLR3 or TLR4 antagonists (see Methods). Statistical significance was calculated using a two-tailed Wilcoxon signed-rank test (**, $p < 0.01$; n = 9).
