# Supplementary material for: A Reproducibility-Based Computational Framework Identifies an Inducible, Enhanced Antiviral State in Dendritic Cells from HIV-1 Elite Controllers
Source: Genome Biol. 2018 Jan 29;19:10. doi: 10.1186/s13059-017-1385-x (PMC5789701; doi:10.1186/s13059-017-1385-x)
Supplement: Supplementary file 7 — AOM. Additional online materials. (PDF 243 kb) [file 13059_2017_1385_MOESM7_ESM.pdf]

Additional Online Methods (AOM) for:

A Reproducibility-Based Computational Framework Identifies An Inducible, Enhanced Antiviral Dendritic Cell State In HIV-1 Elite Controllers

Enrique Martin-Gayo[1,†], Michael B. Cole[2,†], Kellie E. Kolb[1,3,4,†], Zhengyu Ouyang[1], Jacqueline Cronin[1], Samuel W. Kazer[1,3,4], Jose Ordovas-Montanes[1,3,4], Mathias Lichterfeld[1,5], Bruce D. Walker[1,6], Nir Yosef[1,7,8,*], Alex K. Shalek[1,3,4,*], Xu G. Yu[1,5,*]

[1] Ragon Institute of MGH, MIT and Harvard, Cambridge, MA

[2] Department of Physics, University of California, Berkeley, Berkeley, CA

[3] Institute for Medical Engineering & Science (IMES) and Department of Chemistry, MIT, Cambridge, MA

[4] Broad Institute of MIT and Harvard, Cambridge, MA

[5] Infectious Disease Division, Brigham and Women's Hospital, Boston, MA

[6] Howard Hughes Medical Institute, Chevy Chase, MD

[7] Electrical Engineering & Computer Sciences, UC Berkeley, Berkeley, CA

[8] Chan Zuckerberg Biohub Investigator

† These authors contributed equally to this work

* These senior authors contributed equally to this work

* To whom correspondence should be addressed: niryosef@berkeley.edu (NY), shalek@mit.edu (AKS), xyu@mgh.harvard.edu (XY)

APPENDIX - Extending Irreproducible Discovery Rate (IDR) Model Inference to m Sample Replicates

Extending Irreproducible Discovery Rate (IDR) Model Inference to m Sample Replicates

1 Covariance Matrix, Determinant, and Inverse

In section 4 of the Supplementary Materials for Li et el. 2011[24], "Extension of our model to the case of $m > 2$." the authors present their model extended to m-replicates. For each component, $k = 0, 1$, the m-dimensional covariance matrix can be written:

$$\Sigma_k = \sigma_k^2 \left[(1 - \rho_k) I + \rho_k \mathbb{1} \right] \tag{1}$$

Below we will briefly consider properties of this matrix.

1.1 Covariance Matrix Determinant

The matrix determinant can be computed via Sylvester's determinant theorem:

$$\begin{aligned} |\Sigma_k| &= \left| \sigma_k^2 \left[(1 - \rho_k) I + \rho_k \mathbb{1} \right] \right| \\ &= \sigma_k^{2m} (1 - \rho_k)^m \left| I + \frac{\rho_k}{1 - \rho_k} \mathbb{1} \right| \\ &= \sigma_k^{2m} (1 - \rho_k)^m \left(1 + \frac{\rho_k}{1 - \rho_k} m \right) \\ &= \sigma_k^{2m} (1 - \rho_k)^{m-1} (1 + (m - 1) \rho_k) \end{aligned} \tag{2}$$

1.2 Covariance Matrix Inverse

The matrix inverse can be computed via the Sherman-Morrison formula:

$$\begin{aligned}
\Sigma_k^{-1} &= \left(\sigma_k^2 \left[(1-\rho_k) I + \rho_k \mathbb{1}\right]\right)^{-1} \\
&= \frac{1}{\sigma_k^2 (1-\rho_k)} \left(I + \frac{\rho_k}{1-\rho_k}\mathbb{1}\right)^{-1} \\
&= \frac{1}{\sigma_k^2 (1-\rho_k)} \left(I - \frac{\frac{\rho_k}{1-\rho_k}\mathbb{1}}{1 + m\frac{\rho_k}{1-\rho_k}}\right) \\
&= \frac{1}{\sigma_k^2 (1-\rho_k)} \left(I - \frac{\rho_k}{1+(m-1)\rho_k}\mathbb{1}\right)
\end{aligned} \tag{3}$$

2 Maximum Log-Likelihood of Pseudo-Data

Considering the general model above, we can extend Equation (1.5) from section 1 of the Supplementary Materials for Li et el. 2011[24], "Estimation algorithm for the copula mixture model." This equation represents the second term of $Q(\theta, \theta^t)$:

$$\begin{aligned}
El_z = \sum_{i=1}^{n} EK_i \Bigg\{ & \log\left(\frac{1}{\sqrt{(2\pi)^m \sigma_1^{2m} (1-\rho_1)^{m-1} (1+(m-1)\rho_1)}}\right) \\
& - \frac{1}{2\sigma_1^2 (1-\rho_1)} \sum_{p,q}^{m} \left\{ (z_{i,p}-\mu_1)(z_{i,q}-\mu_1)\left(I_{pq} - \frac{\rho_1}{1+(m-1)\rho_1}\mathbb{1}_{pq}\right) \right\} \Bigg\}
\end{aligned} \tag{4}$$

As outlined in that section, we can obtain estimates for model parameters (μ_1, σ_1, and ρ_1) by maximizing the expected likelihood.

2.1 μ_1 Derivative and MLE

Taking derivatives w.r.t. the mean paramater, μ_1, we have the following:

$$\frac{\partial l_z}{\partial \mu_1} = \sum_{i=1}^{n} K_i \left\{ \frac{1}{2\sigma_1^2 (1-\rho_1)} \sum_{p,q}^{m} \left\{ (z_{i,p} + z_{i,q} - 2\mu_1)\left(I_{pq} - \frac{\rho_1}{1+(m-1)\rho_1}\mathbb{1}_{pq}\right) \right\} \right\} \tag{5}$$

Setting the right-hand to zero (assuming $\sigma_1 > 0$, $\rho_1 < 1$, and $m > 0$):

$$0 = \sum_{i=1}^{n} K_i \left(\overline{z_i} - \mu_1\right) \tag{6}$$

Solving for K_i, we see that the MLE estimate of the mean is a weighted mean of replicate means:

$$\mu_1^{(t+1)} = \frac{\sum_{i=1}^n K_i^{(t+1)} \overline{z_i}}{\sum_{i=1}^n K_i^{(t+1)}} \tag{7}$$

2.2 σ_1 Derivative

Taking derivatives w.r.t. the standard deviation, σ_1, we have the following:

$$\frac{\partial l_z}{\partial \sigma_1} = \sum_{i=1}^n K_i \Bigg\{ -\frac{m}{\sigma_1} + \frac{1}{\sigma_1^3 (1-\rho_1)} \sum_{p,q}^m \Big\{ (z_{i,p} - \mu_1)(z_{i,q} - \mu_1) \Big(I_{pq} - \frac{\rho_1}{1+(m-1)\rho_1} \mathbb{1}_{pq} \Big) \Big\} \Bigg\} \tag{8}$$

Setting the right-hand to zero (assuming $\sigma_1 > 0$, and $m > 0$):

$$0 = \sum_{i=1}^n K_i \Bigg\{ -\sigma_1^2 + \frac{1}{m(1-\rho_1)} \sum_{p,q}^m \Big\{ (z_{i,p} - \mu_1)(z_{i,q} - \mu_1) \Big(I_{pq} - \frac{\rho_1}{1+(m-1)\rho_1} \mathbb{1}_{pq} \Big) \Big\} \Bigg\} \tag{9}$$

2.3 ρ_1 Derivative

Taking derivatives w.r.t. the correlation coefficient, ρ_1, we have the following:

$$\begin{aligned} \frac{\partial l_z}{\partial \rho_1} = \sum_{i=1}^n EK_i \Bigg\{ & \frac{1}{2} \Big\{ \frac{m-1}{1-\rho_1} - \frac{m-1}{1+(m-1)\rho_1} \Big\} \\ & - \frac{1}{2\sigma_1^2 (1-\rho_1)^2} \sum_{p,q}^m \Big\{ (z_{i,p} - \mu_1)(z_{i,q} - \mu_1) \Big(I_{pq} - \frac{\rho_1}{1+(m-1)\rho_1} \mathbb{1}_{pq} \Big) \Big\} \\ & + \frac{1}{2\sigma_1^2 (1-\rho_1)} \sum_{p,q}^m \Big\{ (z_{i,p} - \mu_1)(z_{i,q} - \mu_1) \Big(\frac{1}{(1+(m-1)\rho_1)^2} \mathbb{1}_{pq} \Big) \Big\} \Bigg\} \end{aligned} \tag{10}$$

Setting the right-hand to zero (assuming $\sigma_1 > 0$, and $\rho_1 < 1$):

$$\begin{aligned} 0 = \sum_{i=1}^n EK_i \Bigg\{ & \Big\{ \frac{1}{1-\rho_1} - \frac{1}{1+(m-1)\rho_1} \Big\} (m-1)\sigma_1^2 (1-\rho_1)^2 \\ & - \sum_{p,q}^m \Big\{ (z_{i,p} - \mu_1)(z_{i,q} - \mu_1) \Big(I_{pq} - \frac{\rho_1}{1+(m-1)\rho_1} \mathbb{1}_{pq} \Big) \Big\} \\ & + \sum_{p,q}^m \Big\{ (z_{i,p} - \mu_1)(z_{i,q} - \mu_1) \Big(\frac{1-\rho_1}{(1+(m-1)\rho_1)^2} \mathbb{1}_{pq} \Big) \Big\} \Bigg\} \end{aligned} \tag{11}$$

2.4 MLEs for σ_1 and ρ_1

In order to solve the system of equations (9) and (11) above we define a weighted covariance, C, and a total weight, W:

$$\begin{aligned} C_{pq}^{(t+1)} &= \sum_{i=1}^{n} K_i^{(t+1)} \left(z_{i,p} - \mu_1^{(t+1)}\right)\left(z_{i,q} - \mu_1^{(t+1)}\right) \\ W^{(t+1)} &= \sum_{i=1}^{n} K_i^{(t+1)} \end{aligned} \tag{12}$$

The trace and sum of C are important data summaries:

$$\begin{aligned} T^{(t+1)} &= \sum_{p,q}^{m} C_{pq}^{(t+1)} I_{pq} \\ S^{(t+1)} &= \sum_{p,q}^{m} C_{pq}^{(t+1)} \mathbb{1}_{pq} \end{aligned} \tag{13}$$

These definitions allow us to separate variance and correlation terms in equations (9) and (11):

$$\sigma_1^{2(t+1)} = \frac{1}{W^{(t+1)} m \left(1 - \rho_1^{(t+1)}\right)} \left\{ T^{(t+1)} - \frac{\rho_1^{(t+1)}}{1 + (m-1)\,\rho_1^{(t+1)}} S^{(t+1)} \right\} \tag{14}$$

$$\begin{aligned} \sigma_1^{2(t+1)} = & \frac{1}{W^{(t+1)} \left\{ \frac{1}{1-\rho_1^{(t+1)}} - \frac{1}{1+(m-1)\rho_1^{(t+1)}} \right\} (m-1) \left(1 - \rho_1^{(t+1)}\right)^2} \\ & \times \left\{ T^{(t+1)} - \frac{\rho_1^{(t+1)}}{1 + (m-1)\,\rho_1^{(t+1)}} S^{(t+1)} - \frac{1 - \rho_1^{(t+1)}}{\left(1 + (m-1)\,\rho_1^{(t+1)}\right)^2} S^{(t+1)} \right\} \end{aligned} \tag{15}$$

By equating the right-sides of equations (14) and (1) we obtain a simple expression for the MLE correlation coefficient:

$$\rho_1^{(t+1)} = \frac{S^{(t+1)} - T^{(t+1)}}{T^{(t+1)}\,(m-1)} \tag{16}$$

Plugging this expression into the variance equation (14) we obtain a simple expression for MLE variance as well:

$$\sigma_1^{2(t+1)} = \frac{T^{(t+1)}}{W^{(t+1)} m} \tag{17}$$
